# A Mini-Review on the Epidemiology of Canine Parvovirus in China

**DOI:** 10.3389/fvets.2020.00005

**Published:** 2020-02-20

**Authors:** Shanshan Qi, Jianjun Zhao, Donghua Guo, Dongbo Sun

**Affiliations:** College of Animal Science and Veterinary Medicine, Heilongjiang Bayi Agricultural University, Daqing, China

**Keywords:** canine parvovirus, epidemiology, prevalence, coinfection, phylogenetic analysis

## Abstract

Canine viral diarrhea is a severe disease in dogs worldwide. The role of canine parvovirus (CPV) in canine viral diarrhea is a common health problem in dogs, attracting major concern from veterinarians and dog owners across China. In this mini-review, we summarize the CPV epidemiology in China, including its origin, prevalence, coinfection, and the genetic evolution of the virus. The review reveals the correlation between CPV-2 infection and seasonality, a dog's age/gender/breed/vaccination; that CPV-2 is the main causative agent of canine diarrhea in Northeast China and that coinfection with other pathogens is a common occurrence; the predominant CPV epidemic strains were the new CPV-2a, and CPV-2c has shown significant growth trends since 2010. This mini-review will provide valuable information for CPV infections across China and other countries.

## Introduction

In canine disease, viral diarrhea has a high incidence because of etiology complexity, which causes serious harm to the canine industry and dogs. At present, several viral pathogens are related to canine diarrhea in China, including canine parvovirus (CPV) (1, 2), canine coronavirus (CCoV), canine bocavirus, canine kobuviruses (CaKVs), and canine distemper virus (CDV). CPV is an important cause of mortality and morbidity in dogs, especially puppies, in China and the rest of the world (3–5). Variation, recombination, and coinfection have been shown to aggravate clinical symptoms and challenge the prevention and control of CPV infections (6–9). Dogs that become infected by CPV show illness within 3–7 days, presenting with severe gastroenteritis, lethargy, vomiting, fever, and diarrhea (usually bloody) (10–12).

CPV belongs to the genus *Parvovirus*, family *Parvoviridae*, and causes a highly contagious and fatal disease in dogs (1). The original viral strain, designated as CPV-2 to distinguish it from CPV-1 which is also known as canine minute virus and was believed to be non-pathogenic until 1992 (13). CPV-2 is a non-enveloped DNA virus with a linear single-stranded DNA genome (5.2 kb), containing two major open reading frames (ORFs). One ORF encodes the two non-structural proteins (NS1 and NS2), and the other encodes the two capsid proteins (VP1 and VP2) (14). The VP2 protein of CPV-2 is known to affect antigenic properties, playing important roles in controlling viral host ranges and tissue tropisms (15–17).

The main method for controlling the virus in domestic animals is by vaccination, antibody therapy, and traditional Chinese medicine therapy (18, 19). However, the virus is widely distributed in nature, and the morbidity and mortality of CPV-2-infected animals remain high (20). Furthermore, because of vaccine formulations, a dramatic increase in the number of dog or other factors potentially promote the spread of different CPV-2 antigenic variants, increasing disease complexity (21–23). In previous studies, researchers have investigated CPV-2 genetic evolution (5, 24–27), providing important reference information for the prevention and control of the CPV-2 infections. However, the molecular epidemiology and genetic diversity of CPV-2 need to be updated in China. In this review, we have summarized contemporary data on the progression of CPV-2 epidemiology in China, including the virus origin, prevalence, coinfection, and evolution. The aim is to unravel CPV-2 epidemiology and provide new information on virus infections, not only for Chinese dogs and their owners but also for all dog owners across the world.

## The Origin of CPV-2 in China

In 1978, CPV was first reported as a viral diarrhea pathogen in canine populations in the United States (2) and other countries (28). Since 1970, suspected CPV-2 infections have repeatedly caused acute diarrhea disease in police dogs in East, Southwest, and Northeast China (29). However, due to a lack of diagnostic methods, the causative agents of this disease were not confirmed as CPV-2 until 1982 (30). In 1983, a series of diagnostic methods, including hemagglutination tests, hemagglutination inhibition tests, electron microscopy, and immunoelectron microscopy confirmed that CPV-2 was the causative agent of diarrhea in dogs (29, 31, 32). Since then, comprehensive CPV-2 epidemiological, diagnostic, and vaccine development research has continued across China (33–35). Subsequently, CPV-2 has gradually become one of the most important pathogens of viral diarrhea in Chinese canine populations, with a high prevalence.

## The Prevalence of CPV-2 in China

### Morbidity and Mortality of CPV-2 in China

**Figure 1:**
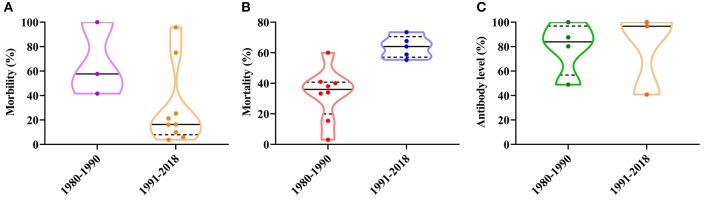
**(A)** Temporal distribution analysis of the morbidity of canine parvovirus (CPV) in China. **(B)** Temporal distribution analysis of the mortality of CPV in China. **(C)** Temporal distribution analysis of antibody levels of CPV in China.

Since CPV-2 was confirmed in China, the disease has shown a local endemic tendency, with different morbidities and mortalities. In the 1970s, CPV-2 morbidity varied between 30 and 40%, with a mortality rate of over 10% (29). In the 1980s, CPV-2 incidence varied between 41.61 and 100%, and the mortality rate varied from 3.1 to 60% (33, 36). The positive rate of expression of CPV-2 antibody varied from 48.92 to 100% in the 1980s (34, 37). From the 1990s to the current era, CPV-2 incidence in clinical animal hospitals has varied between 3.90 and 95.8%, and the mortality rate has varied between 20.17 and 73.47% (38–47). The positive rate of expression of CPV-2 antibody varied from 40.9 to 100% (48, 49) (Figure 1). Since the discovery of the virus, the overall morbidity has been reduced and antibody levels to CPV-2 have remained stable; however, animal mortality rates have significantly increased, suggesting that the virus is significantly more virulent and more destructive to the animal, may be due in large part to emerging CPV antigenic variants (Figure 1).

### Seasonality of CPV-2 Infections

**Figure 2:**
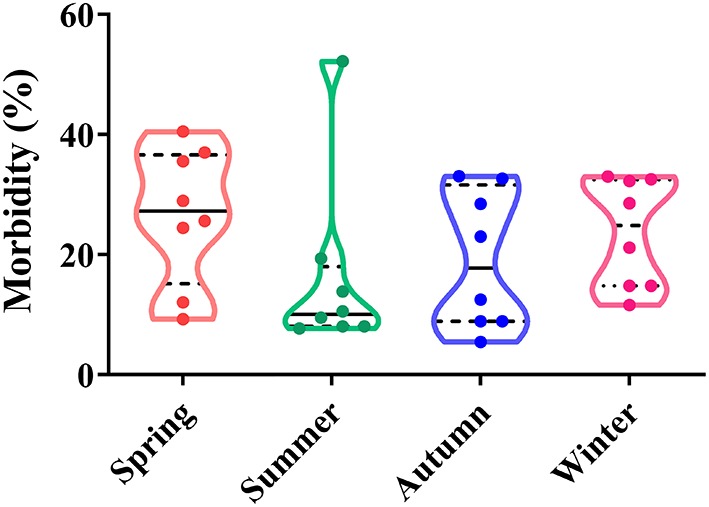
Seasonal correlation analysis of canine parvovirus (CPV) infections in China.

**Figure 3:**
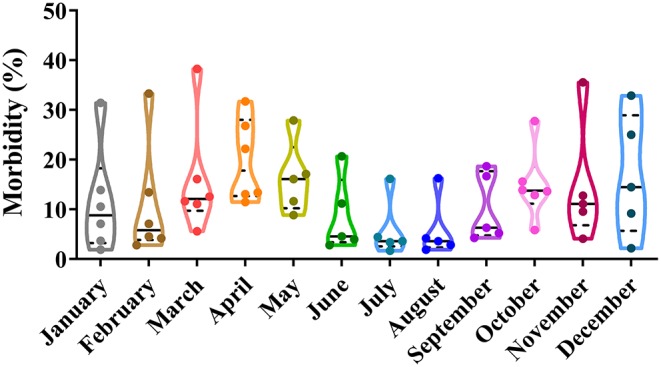
Monthly correlation analysis of canine parvovirus (CPV) infections in China.

Depending on seasonal variation, the occurrence of CPV-2 is a year-round phenomenon. In spring (March to May), CPV-2 incidences vary from 9.26 to 40.52%, whereas in summer (June to August), incidences vary from 7.70 to 52.22%; in the autumn (September to November), incidences vary from 5.48 to 33.06%, and in the winter (December to February), they vary from 14.80 to 33.04% (43, 45, 50–54) (Figure 2). The proportion of CPV-2 incidences from January to December varied from 1.9 to 31.43%, 2.8 to 33.33%, 5.6 to 38.29%, 11.48 to 31.71%, 8.84 to 27.91%, 2.8 to 20.69%, 1.7 to 16.13%, 1.9 to 16.27%, 4.26 to 18.70%, 5.86 to 27.78%, 4.10 to 35.56%, and 2.20 to 32.88%, respectively (39, 41, 47, 55, 56) (Figure 3). As can be showed from Figures 2, 3, the morbidities in spring, late autumn, and early winter were relatively higher than those in other seasons, which in turn may have been related to large seasonal diurnal temperature differences and changeable climates during these seasons. Several studies have reported that CPV-2 infection rates were higher in spring and autumn (8). Such findings were possibly due to the increased frequency of outdoor activities for dogs and humans, where dogs were more likely to contact with viral pathogens, making them susceptible to disease (41, 42). In general, CPV-2 can cause infections throughout the year and variations in morbidity according to the season in different regions of China, in which the infection is more serious in the spring, late autumn, and early winter.

### Correlations Between a Dog's Age and CPV-2 Infections

**Figure 4:**
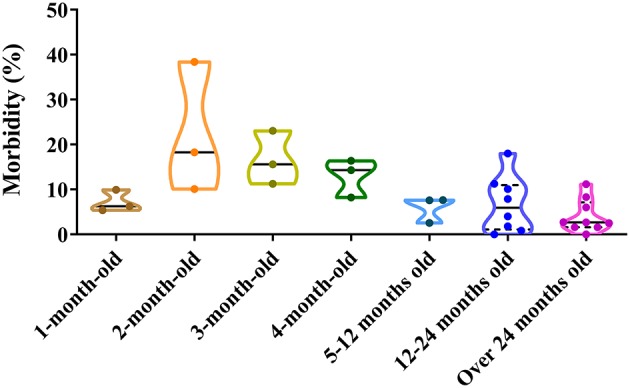
Correlation between a dog's age and canine parvovirus (CPV) infection in China.

Dogs of all ages can be infected by CPV-2. The positive rate of CPV-2 varies by dogs ages. For 1 month old, the positive proportion varies from 5.40 to 9.93%; for 2 months old, it varies from 10.11 to 38.40%; for 3 months old, it varies from 11.26 to 23.08%; for 4 months old, it varies from 8.21 to 16.38%; between 5 months and 1 year, it varies from 2.55 to 7.65%; from 1 to 2 years, it varies from 0.00 to 18.03%; and over 2 years old, it varies from 0.00 to 11.20% (39, 41, 42, 46, 47, 50, 55–60) (Figure 4). The figure demonstrated that higher positive rates of CPV-2 were found in animals ranging from 2 to 4 months old, which was similar to the observations by Cavalli et al. (59) and Geng et al. (60). The low morbidity in dogs aged <1 month old most likely resulted from acquired maternal antibody, while the low morbidity in dogs aged more than 4 months old most likely resulted from the development of adaptive immune responses; dogs at 2–4 months account for a high CPV-2 incidence, which may reflect decreases in dog-specific maternal antibody levels (8, 56, 61, 62). Adult dogs are relatively resistant to the virus; this may be related to increased vaccination rates and developed immune functions, thereby reducing disease incidence (53). In conclusion, there are significant differences in CPV-2 susceptibilities at different dog ages, with a negative correlation between the incidence of CPV-2 and dog over 2 months old (Figure 4).

### Correlations Between a Dog's Gender and CPV-2 Infections

**Figure 5:**
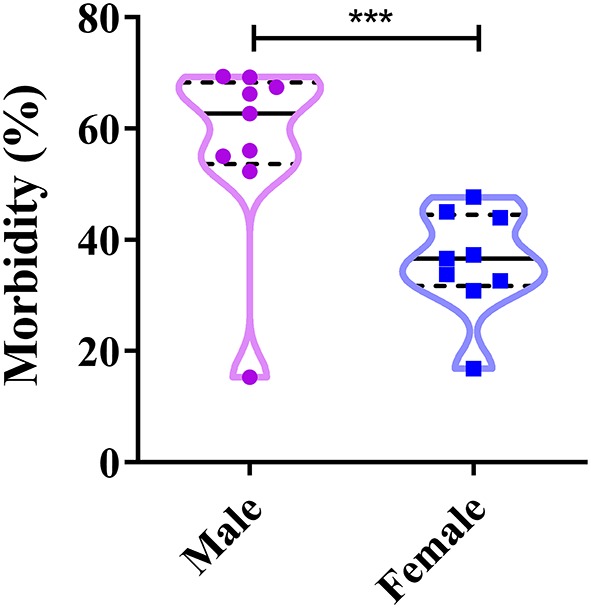
Correlation between a dog's gender and canine parvovirus (CPV) infection in China. ***P < 0.05.

The proportion of CPV-positive male dogs varies between 15.29 and 69.20%, whereas that of positive female dogs varies between 16.80 and 47.7% (39, 41–43, 48, 51, 52, 54) (Figure 5). The figure suggests that both male and female dogs can be infected with CPV-2; however, male dogs account for the higher disease prevalence (8, 63). This appears to be related to the dog market in China; most pet stores predominantly sell male dogs, as the breeding number of male dogs is higher than that for female dogs, which leads to higher infection rates in these male dogs (40, 58). Nevertheless, the prevalence of CPV-2 in dogs shows significant gender variation, usually occurring more in male dogs than female dogs (Figure 5).

### Correlations Between the Breed of Dog and CPV-2 Infections

**Figure 6:**
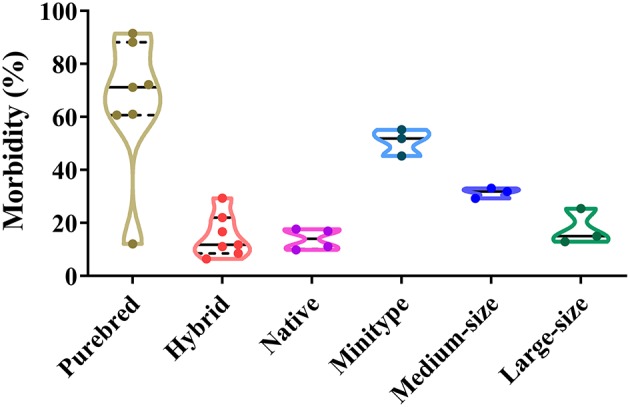
Correlation between dog breeds and canine parvovirus (CPV) infection in China.

In total, different dog breeds can be infected by CPV-2 (45, 60). The proportion of CPV-2 positive purebreds, hybrids, and native dogs varies from 12.15 to 91.5%, 6.46 to 29.40%, and 9.90 to 17.7%, respectively. The proportion of CPV-2 positive mini-types, medium-sized, and large-sized dogs varies from 45.26 to 55.18%, 29.31 to 33.10%, and 12.92 to 25.43%, respectively (39, 42, 45, 47, 50–52, 58) (Figure 6). Among confirmed CPV-2 cases, the incidence of disease in purebred dogs was the highest due to increasing numbers of purebred dogs being sought by owners, which was far higher than hybrids and native dogs (Figure 6). In addition, hybrids and native dogs have greater resistance to CPV, and importantly, they were able to better adapt to local climates and environments (58). The figure showed that all dog breeds are susceptible to the virus; however, hybrids and native dogs are less susceptible than many purebreds, which should be investigated in future studies.

### Correlation Between Dog Vaccination and CPV-2 Infection

**Figure 7:**
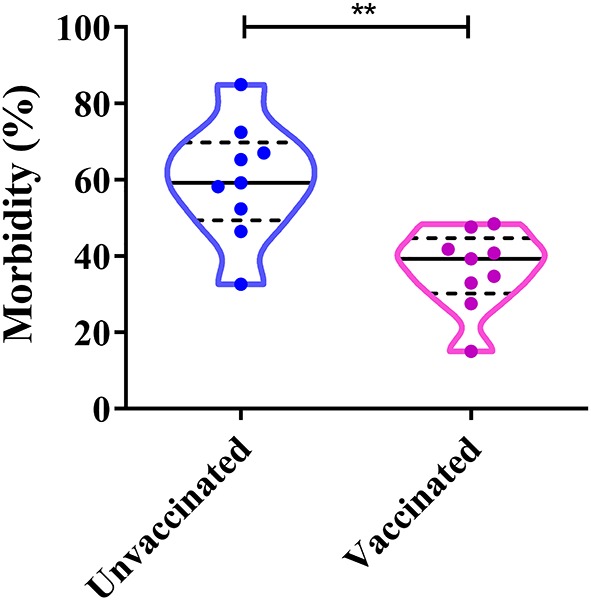
Correlation between dog vaccinations and canine parvovirus (CPV) infection in China. **P < 0.05.

The proportion of CPV-2-positive unvaccinated dogs varies between 32.63 and 84.98%, whereas CPV-2 positive vaccinated dogs (vaccinated at least once) varies between 15.02 and 48.42% (Figure 7). Both vaccinated and unvaccinated dogs can be affected by CPV-2 (60). The positive rates of CPV in unvaccinated dogs were significantly higher than those in vaccinated animals (41–43, 47, 50, 55, 56, 58, 60). The above studies show that vaccination is vitally important for the prevention and control of CPV-2. In China, insufficient attention is often paid to dogs, and therefore, individuals have not yet realized the importance of immunizing their pets. This in turn leads to a large proportion of unimmunized sick dogs. In conclusion, the key to preventing canine parvovirus is to establish a timely and functional immune response.

### Recovery Rates of CPV-2 Affected Dogs

Fluid replacement, systemic antibiotic administration, antinausea medicines, antidiarrhea medicines, and a rigorous diet combined with monoclonal antibodies are the main treatment methods for CPV-2-infected dogs; however, the recovery rates vary from 27.8 to 93.5% (39, 53, 55, 56, 64, 65). Both disease and pathology of the infected animal differ depending on the age. CPV-2 infection in adult dogs results in temporary panleukopenia or lymphopenia; CPV-2 infection in neonatal animals causes myocarditis (12, 66). CPV-2 monoclonal antibodies are highly therapeutic in a short period of time and have treatment well effect (53). The cure rate for a CPV-2 single infection is higher than that of a coinfection with other viruses (41). With improvements in medical treatments, CPV-2 cure rates have been improved using specific drugs or other treatments.

### Coinfection by CPV-2 With Other Pathogens

**Figure 8:**
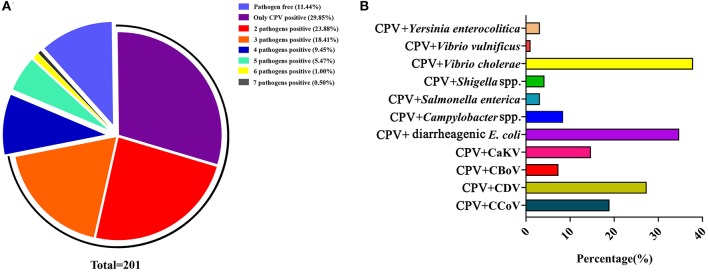
**(A)** Coinfection analysis of CPV with multiple pathogens. **(B)** The coinfection rate of CPV with other enteric viruses or bacteria. The data were cited from previously published papers (60, 67–71) in our lab.

The etiology of canine diarrhea is extremely complex because of the frequency of coinfections. In our previous study, we detected and analyzed viruses and bacteria from 201 diarrhea dog feces samples, collected from three cities in Heilongjiang province, Northeast China. We detected CPV-2, CCoV, CaKV, CBV, and CDV, and we also detected diarrheagenic *Escherichia coli, Campylobacter* spp., *Salmonella enterica, Shigella* spp., *Vibrio cholerae, Vibrio vulnificus*, and *Yersinia enterocolitica* (60, 67–71). Of the 201 fecal samples, 11.44% (23/201) were pathogen free and 88.56% (178/201) were positive for one or more pathogen (virus or bacteria). Among these, CPV-2 only infected 29.85% (60/201) of samples, whereas coinfection of two-, three-, four-, five-, six-, and seven-pathogen-positive samples accounted for 23.88% (48/201), 18.41% (37/201), 9.45% (19/201), 5.47% (11/201), 1.00% (2/201), and 0.50% (1/201), respectively (Figure 8A). Of the 201 samples, the CPV-2-positive rate was 47.26% (95/201). The coinfection rates with CPV-2 occurred with the following 11 pathogens: CCoV, CDV, canine bocavirus, CaKV, diarrheagenic *E. coli, Campylobacter* spp., *S. enterica, Shigella* spp., *V. cholerae, V. vulnificus*, and *Y. enterocolitica*, at frequencies of 18.95, 27.37, 7.37, 14.74, 34.74, 8.42, 3.16, 4.21, 37.89, 1.05, and 3.16%, respectively (60, 67–71) (Figure 8B). These data indicated that CPV-2 was the main causative agent of canine diarrhea in Northeast China and that coinfection with other pathogens was a common occurrence. Zhao et al. (8) reported that the coinfection rate of CPV-CDV, CPV-CCoV, CPV coccidium (*Isospora*), CPV hookworm (*Ancylostoma*), CPV roundworm (*Toxocara*), CPV tapeworm (*Dipylidium*), and CPV *Babesia* spp. was 4.79% (56/1,169), 1.11% (13/1,169), 10.00% (117/1,169), 2.40% (28/1,169), 1.03% (12/1,169), 0.17% (2/1,169), and 0.09% (1/1,169), respectively. Detection of the virus was conducted using a CPV, CDV, or CCoV colloidal gold test strip, respectively; detection of the parasite was conducted by microscopy according to the characteristic of these parasites; and detection of the bacteria was conducted by PCR (8). The coinfection of canine enteric viruses and bacteria frequently occur in diarrheic dogs, both in China and other countries (26, 59, 73). The potential pathogenic agents were complicated, and there are severe coinfection events of canine diarrhea in China. CPV infection occurs in both unvaccinated and vaccinated dogs (72). In addition, some studies suggested that dogs harboring parasites were especially susceptible to CPV infection (74, 75). The accompanying pathogens that occur with CPV-2 coinfections suggest that they should be considered in vaccination programs to control CPV-2 outbreaks (76).

## Genetic Evolution of CPV in China

### Subtyping CPV Strains

**Figure 9:**
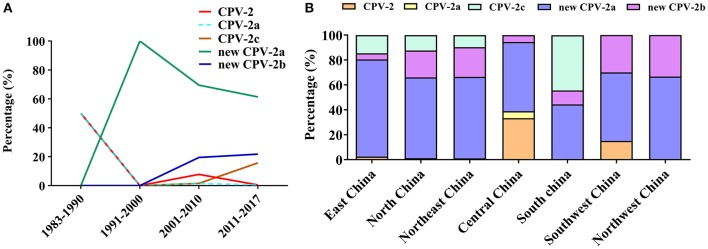
**(A)** Temporal distribution analysis of canine parvovirus (CPV) subtypes in China. **(B)** Geographical distribution analysis of CPV subtypes in China.

The CPV is undergoing positive selection and has evolved independently in different populations (77), in which genomic substitution rates were similar to those of RNA viruses (66). So far, CPV circulating antigenic variants include CPV-2, CPV-2a, CPV-2b, CPV-2c, new CPV-2a, and the new CPV-2b (24). To clarify the evolution of CPV strains in China, we analyzed the geographical and temporal distribution of CPV variants in China collected from GenBank (Figure 9). In the early 1980s, the original CPV-2 started circulating in Chinese canine populations; however, in 1986, CPV-2a replaced CPV-2 as the predominant Chinese isolate (78). The figure has shown that the new CPV-2a has gradually become stable in recent years, while the new CPV-2b has increased slowly, and the CPV-2c has shown significant growth trends since 2010 (Figure 9A). In addition, new CPV-2a and CPV-2b strains are distributed over all parts of the country; CPV-2c appears to have circulated in the eastern, northern, northeastern, and southern regions of China. CPV-2 circulated in eastern, central, and southwestern regions, whereas CPV-2a circulated only in Central China (Figure 9B). In their work, Zhang et al. (20) indicated that new CPV-2a and CPV-2b strains started circulating in China from the late 1990s onwards and that new CPV-2a has become the predominant CPV type, which is consistent with our data (79) (Figure 9B). The new CPV-2a and CPV-2b strains have been cocirculating in Northeast China, which is consistent with previous observations (58). In conclusion, since the first outbreak of CPV-2 in China, the predominant CPV-2 epidemic strains were the new CPV-2a that is cocirculating with the new CPV-2b and CPV-2c. The relative frequencies of these strains appear to differ by geographic regions (80, 81). The detailed information of CPV-2 strains used in temporal and geographical distribution analysis was showed in Supplementary Materials.

### Phylogenetic Analysis of CPV Strains in China

**Figure 10:**
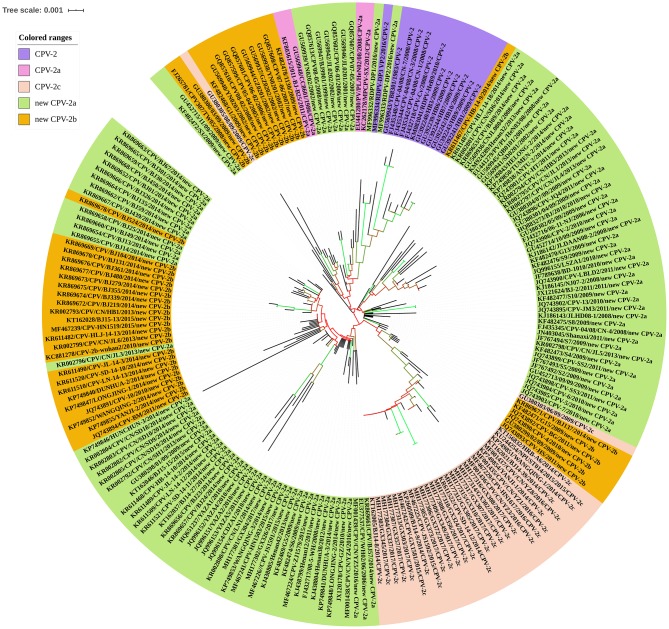
Phylogenetic analysis of canine parvovirus (CPV) strains based on VP2 gene sequences.

The CPV was characterized by strong selection for specific mutations in VP2, the driving forces of which most likely was optimal receptor binding and antigenic escape (82). To further explore the evolution of CPV, the entire VP2 gene sequences of CPV strains were retrieved from the NCBI nucleotide database to construct phylogenetic trees. These nucleotide sequences were used to generate a neighbor-joining phylogenetic tree using the ClustalX alignment tool in the MEGA6.06 software program (83). Neighbor-joining phylogenetic trees were constructed using the p-distance model, with 1,000 bootstrap replicates, and the remaining default parameters in the MEGA 6.06 software. The phylogenetic tree was annotated with the Interactive Tree of Life (iTOL) software (http://itol.embl.de/), an online tool for the display and annotation of phylogenetic trees (84). A VP2-gene-based phylogenetic analysis revealed that the 204 CPV strains clustered into their respective antigenic variants: CPV-2, CPV-2a, CPV-2c, new CPV-2a, and new CPV-2b, all circulating in different Chinese provinces or municipalities. In this analysis, the new CPV-2a strains displayed wider distributions and had a greater number. The CPV-2a and CPV-2c strains have been sporadically detected. Interestingly, the new CPV-2b strain was dispersed into the evolutionary topological branches of the new CPV-2a strain (Figure 10). In conclusion, circulating CPV antigenic variants in China include CPV-2, CPV-2a, CPV-2c, new CPV-2a, and new CPV-2b. A summary of VP2 sequences has provided a comprehensive perspective on CPV-2 evolution. Going forward, correlative biological studies should be performed.

### The Spreading of Global CPV Strains

The global distribution and temporal dynamics of CPV variants have been extensively studied by analyzing CPV strains from diverse geographic regions and different years (66, 82, 85). Recent reports indicated that CPV-2a is prevalent mainly in Australia, India, Hungary, Korea, China, and Greece (79, 80, 86–89). CPV-2b is the prevalent variant in the United States, the United Kingdom, and Japan, but with different frequencies (3, 24, 90, 91). The CPV-2c is detected mainly in Italy and Argentina, and Uruguay (24, 77, 92–95). CPV-2 was completely replaced by the CPV-2a variant in America, which is consistent with the prevalence of CPV in China (96, 97). CPV-2c has reached considerable frequencies in some countries of the America and Europe, which is similar to the prevalence in China (17, 77, 93, 94, 98, 99). Pérez reported that the CPV-2c was the only variant detected in the Uruguayan dog population from 2007 to 2009. However, CPV-2a with a relatively high prevalence replacing an established CPV-2c in 2010 (94). Interestingly, similar studies indicated that the CPV-2a was introduced to South America, which was associated with strengthening of connections between China and Uruguay (100). The migrations of CPV are likely spread among countries in close geographic proximity through the movement of infected animals or mechanical vectors (82). Nevertheless, CPV-2a, 2b, and 2c are circulating in almost equal proportions in Tunisia (101). CPV-2a, CPV-2b, and CPV-2c are currently spreading globally, and their relative frequencies may be related to the geographic region and time of the sample collection and different commercial flows of dogs imported from foreign countries (3, 77)

## Prospects

In conclusion, we have summarized novel epidemiological information for CPV infections in China, including the origin, prevalence, and genetic evolution of the virus. This mini-review will facilitate an increased comprehension of CPV strains in China. Tracing virus mutations, developing effective vaccines, enhancing quarantine measures, and developing antibodies against CPV-2 antigenic variants will provide effective measures in preventing and controlling CPV-2 infections in the future. Furthermore, pathogenicity differences between different CPV-2 antigenic variants, and the cross-protection effects of existing commercial vaccines against CPV-2 are the important problems that require attention.

## Author Contributions

DS conceived the study. SQ, DG, and JZ analyzed the data. DS and SQ wrote the manuscript.

### Conflict of Interest

The authors declare that the research was conducted in the absence of any commercial or financial relationships that could be construed as a potential conflict of interest.
