# Supplementary material for: A Mini-Review on the Epidemiology of Canine Parvovirus in China
Source: Front Vet Sci. 2020 Feb 20;7:5. doi: 10.3389/fvets.2020.00005 (PMC7044151; doi:10.3389/fvets.2020.00005)
Supplement: Supplementary file 1 [file Data_Sheet_1.docx]

**TABLE S1.** Temporal distribution analysis of the genotypes of CPV-2 in China.

| Year | CPV-2 | CPV-2a | CPV-2b | new CPV-2a | new CPV-2b | CPV-2c |
| --- | --- | --- | --- | --- | --- | --- |
| 1983 | 1 | 0 | 0 | 0 | 0 | 0 |
| 1986 | 0 | 1 | 0 | 0 | 0 | 0 |
| 1999 | 0 | 0 | 0 | 2 | 0 | 0 |
| 2001 | 0 | 1 | 0 | 1 | 0 | 0 |
| 2002 | 0 | 0 | 0 | 3 | 4 | 0 |
| 2005 | 0 | 0 | 0 | 3 | 3 | 0 |
| 2006 | 0 | 0 | 0 | 3 | 1 | 0 |
| 2007 | 0 | 0 | 0 | 21 | 4 | 0 |
| 2008 | 3 | 1 | 0 | 9 | 2 | 0 |
| 2009 | 6 | 0 | 0 | 22 | 5 | 2 |
| 2010 | 1 | 0 | 0 | 27 | 6 | 0 |
| 2011 | 0 | 1 | 0 | 35 | 7 | 0 |
| 2012 | 0 | 1 | 0 | 12 | 0 | 0 |
| 2013 | 0 | 0 | 0 | 13 | 2 | 0 |
| 2014 | 1 | 0 | 0 | 145 | 66 | 35 |
| 2015 | 0 | 0 | 0 | 23 | 8 | 7 |
| 2016 | 1 | 0 | 0 | 4 | 0 | 4 |
| 2017 | 0 | 0 | 0 | 2 | 0 | 14 |
| Total | 13 | 5 | 0 | 325 | 108 | 62 |

**TABLE S2**. Geographical distribution analysis of the genotypes of CPV-2 in China.

|  | CPV-2 | CPV-2a | CPV-2b | new CPV-2a | new CPV-2b | CPV-2c |
| --- | --- | --- | --- | --- | --- | --- |
| East China | 1 | 0 | 0 | 32 | 2 | 6 |
| North China | 1 | 1 | 0 | 105 | 35 | 20 |
| Northeast China | 1 | 1 | 0 | 121 | 44 | 18 |
| Central China | 6 | 1 | 0 | 10 | 1 | 0 |
| South china | 0 | 0 | 0 | 16 | 4 | 16 |
| Southwest China | 3 | 0 | 0 | 11 | 6 | 0 |
| Northwest China | 0 | 0 | 0 | 2 | 1 | 0 |
| Total | 12 | 3 | 0 | 297 | 93 | 60 |

**TABLE S3.** CPV-2 strains used in geographical distribution analysis.

| GenBank accession no./Name | Isolated region | Isolated time | Genotype |
| --- | --- | --- | --- |
| MF996333.1/RDPV-DP2 | Shandong/ East China | 2016 | new CPV-2a |
| MF996332.1/RDPV-DP1 | Shandong/ East China | 2016 | new CPV-2a |
| GU569941.1/SD0201 | Shandong/ East China | 2002 | new CPV-2a |
| FJ432716.1/06-11-NJ | Jiangsu/ East China | 2006 | new CPV-2a |
| MF001439.1/CPV/CN/YZ5 | Jiangsu/ East China | 2016 | new CPV-2a |
| MF001438.1/CPV/CN/YZ4 | Jiangsu/ East China | 2016 | new CPV-2a |
| MF001437.1/CPV/CN/YZ8 | Jiangsu/ East China | 2016 | CPV-2c |
| MF001436.1/CPV/CN/YZ3 | Jiangsu/ East China | 2016 | CPV-2c |
| MF001435.1/CPV/CN/YZ1 | Jiangsu/ East China | 2016 | CPV-2c |
| KJ186145.1/NJ07-2 | Jiangsu/ East China | 2008 | new CPV-2a |
| KR611522.1/CPV-SD-14-12 | Shandong/ East China | 2014 | CPV-2c |
| KR611521.1/CPV-SD-14-11 | Shandong/ East China | 2014 | new CPV-2a |
| KR611520.1/CPV-SD-14-10 | Shandong/ East China | 2014 | new CPV-2b |
| MF467226.1//CPV-JS1591 | Jiangsu/ East China | 2015 | new CPV-2a |
| MF467225.1/CPV-JS1592 | Jiangsu/ East China | 2015 | CPV-2c |
| MF467224.1/CPV-ZJ1579 | Zhejiang/ East China | 2015 | new CPV-2a |
| MF996334.1/RDPV-DP3 VP2 | Shandong/ East China | 2016 | CPV-2 |
| KP260509.1/BJ14-9 | Beijing/ North China | 2014 | CPV-2c |
| KR869678.1/CPV/BJ524 | Beijing/ North China | 2014 | new CPV-2b |
| KR869677.1/CPV/BJ480 | Beijing/ North China | 2014 | new CPV-2b |
| KR869676.1/CPV/BJ361 | Beijing/ North China | 2014 | new CPV-2b |
| KR869675.1/CPV/BJ355 | Beijing/ North China | 2014 | new CPV-2b |
| KR869674.1/CPV/BJ339 | Beijing/ North China | 2014 | new CPV-2b |
| KR869673.1/CPV/BJ279 | Beijing/ North China | 2014 | new CPV-2b |
| KR869672.1/CPV/BJ219 | Beijing/ North China | 2014 | new CPV-2b |
| KR869671.1/CPV/BJ137 | Beijing/ North China | 2014 | new CPV-2b |
| KR869670.1/CPV/BJ131 | Beijing/ North China | 2014 | new CPV-2b |
| KR869669.1/CPV/BJ104 | Beijing/ North China | 2014 | new CPV-2b |
| KR869668.1/CPV/BJ450 | Beijing/ North China | 2014 | new CPV-2a |
| KR869667.1/CPV/BJ439 | Beijing/ North China | 2014 | new CPV-2a |
| KR869666.1/CPV/BJ326 | Beijing/ North China | 2014 | new CPV-2a |
| KR869665.1/CPV/BJ301 | Beijing/ North China | 2014 | new CPV-2a |
| KR869664.1/CPV/BJ135 | Beijing/ North China | 2014 | new CPV-2a |
| KR869663.1/CPV/BJ67 | Beijing/ North China | 2014 | new CPV-2a |
| KR869662.1/CPV/BJ59 | Beijing/ North China | 2014 | new CPV-2a |
| KR869661.1/CPV/BJ57 | Beijing/ North China | 2014 | new CPV-2a |
| KR869660.1/CPV/BJ49 | Beijing/ North China | 2014 | new CPV-2a |
| KR869659.1/CPV/BJ30 | Beijing/ North China | 2014 | new CPV-2a |
| KR869658.1/CPV/BJ25 | Beijing/ North China | 2014 | new CPV-2a |
| KR869657.1/CPV/BJ24 | Beijing/ North China | 2014 | new CPV-2a |
| KR869656.1/CPV/BJ23 | Beijing/ North China | 2014 | new CPV-2a |
| KR869655.1/CPV/BJ14 | Beijing/ North China | 2014 | new CPV-2a |
| KR869654.1/CPV/BJ13 | Beijing/ North China | 2014 | new CPV-2a |
| KR869653.1/CPV/BJ09 | Beijing/ North China | 2014 | new CPV-2a |
| KR869652.1/CPV/BJ01 | Beijing/ North China | 2014 | new CPV-2a |
| GU569947.1/BJ9901 | Beijing/ North China | 1999 | new CPV-2a |
| KM083041.1/RDPV-HeB(10)9 | Hebei/ North China | 2010 | CPV-2 |
| JF789638.1/BD-1010 | Hebei/ North China | 2010 | new CPV-2a |
| HQ883273.1/BJ/2010 | Beijing/ North China | 2010 | new CPV-2a |
| JX121624.1/BJ-2/2011 | Beijing/ North China | 2011 | new CPV-2a |
| KP260509.1/BJ14-9 | Beijing/ North China | 2014 | CPV-2c |
| KR611468.1/CPV-HB-14-10 | Hebei/ North China | 2014 | new CPV-2a |
| KR611467.1/CPV-HB-14-9 | Hebei/ North China | 2014 | new CPV-2b |
| KT162037.1/BJ14-27 | Beijing/ North China | 2014 | new CPV-2a |
| KT162021.1/BJ14-39 | Beijing/ North China | 2014 | CPV-2c |
| KT162046.1/BJ15-15 | Beijing/ North China | 2015 | new CPV-2a |
| KT162028.1/BJ15-13 | Beijing/ North China | 2015 | new CPV-2b |
| GU569945.1/QB0101 | Jilin/ Northeast China | 2001 | new CPV-2a |
| GU569943.1/YB8301 | Jilin/ Northeast China | 1983 | CPV-2 |
| GU569948.1/CC8601 | Jilin/ Northeast China | 1986 | CPV-2a |
| GU569946.1/JL0201 | Jilin/ Northeast China | 2001 | new CPV-2a |
| GU569942.1/JL0202 | Jilin/ Northeast China | 2002 | new CPV-2a |
| GU569936.1/CNJL0804 | Jilin/ Northeast China | 2008 | new CPV-2a |
| KJ186143.1/JLHD08-1 | Jilin/ Northeast China | 2008 | new CPV-2a |
| KJ186142.1/JLDAAN08-2 | Jilin/ Northeast China | 2008 | new CPV-2a |
| KR611510.1/CPV-LN-14-13 | Liaoning/ Northeast China | 2014 | new CPV-2b |
| KR611509.1/CPV-LN-14-12 | Liaoning/ Northeast China | 2014 | new CPV-2a |
| KR611490.1/CPV-JL-14-3 | Jilin/ Northeast China | 2014 | new CPV-2b |
| KR611489.1/CPV-JL-14-2 | Jilin/ Northeast China | 2014 | new CPV-2a |
| KR611487.1/CPV-HLJ-14-18 | Heilongjiang/ Northeast China | 2014 | new CPV-2a |
| KR611482.1/CPV-HLJ-14-13 | Heilongjiang/ Northeast China | 2014 | new CPV-2b |
| KP749855.1/YANJI-2 | Jilin/ Northeast China | 2014 | new CPV-2b |
| KP749854.1/YANJI-1 | Jilin/ Northeast China | 2014 | CPV-2c |
| KP749853.1/WANGQING-3 | Jilin/ Northeast China | 2014 | new CPV-2a |
| KP749852.1/WANGQING-2 | Jilin/ Northeast China | 2014 | new CPV-2b |
| KP749851.1/WANGQING-1 | Jilin/ Northeast China | 2014 | CPV-2c |
| KP749850.1/TUMEN-2 | Jilin/ Northeast China | 2014 | new CPV-2a |
| KP749848.1/LONGJING-2 | Jilin/ Northeast China | 2014 | new CPV-2a |
| KP749847.1/LONGJING-1 | Jilin/ Northeast China | 2014 | new CPV-2b |
| KP749846.1/HUNCHUN-3 | Jilin/ Northeast China | 2014 | new CPV-2a |
| KP749843.1/HELONG-2 | Jilin/ Northeast China | 2014 | new CPV-2a |
| KP749841.1/DUNHUA-3 | Jilin/ Northeast China | 2014 | new CPV-2a |
| KP749840.1/DUNHUA-2 | Jilin/ Northeast China | 2014 | new CPV-2b |
| KP749838.1/ANTU-2 | Jilin/ Northeast China | 2014 | new CPV-2a |
| JN403045.1/Shanaxi | Shanxi/ North China | 2011 | new CPV-2a |
| GU392244.1/HB7 | Hubei/ Central China | 2009 | CPV-2 |
| GU392243.1/HB9 | Hubei/ Central China | 2009 | CPV-2 |
| GU392242.1/HB10 | Hubei/ Central China | 2009 | CPV-2 |
| GU392241.1/HB1 | Hubei/ Central China | 2009 | CPV-2 |
| GU392240.1/HB3 | Hubei/ Central China | 2009 | CPV-2 |
| GU392239.1/HB6 | Hubei/ Central China | 2009 | CPV-2 |
| MH177317.1/GX307 | Guangxi/ South China | 2017 | CVP-2c |
| MH177316.1/GX308 | Guangxi/ South China | 2017 | CVP-2c |
| MH177315.1/GX310 | Guangxi/ South China | 2017 | CVP-2c |
| MH177314.1/GX302 | Guangxi/ South China | 2017 | CVP-2c |
| MH177313.1/GX303 | Guangxi/ South China | 2017 | CVP-2c |
| MH177312.1/GX305 | Guangxi/ South China | 2017 | CVP-2c |
| MH177310.1/GX324 | Guangxi/ South China | 2017 | CVP-2c |
| MH177309.1/GX349 | Guangxi/ South China | 2017 | CVP-2c |
| MH177308.1/GX356 | Guangxi/ South China | 2017 | CVP-2c |
| MH177307.1/GX357 | Guangxi/ South China | 2017 | CVP-2c |
| MH177306.1/GX372 | Guangxi/ South China | 2017 | CVP-2c |
| MH177305.1/GX373 | Guangxi/ South China | 2017 | CVP-2c |
| MH177304.1/GX337 | Guangxi/ South China | 2017 | CVP-2c |
| MH177303.1/GX345 | Guangxi/ South China | 2017 | CVP-2c |
| MH177302.1/GX326 | Guangxi/ South China | 2017 | new CPV-2a |
| MH177301.1/GX304 | Guangxi/ South China | 2017 | new CPV-2a |
| EU441279.1/PV/PL/HeN03/08 | Henan/ Central China | 2008 | new CPV-2a |
| EU377537.1/CPV/WH02/06 | Hubei/ Central China | 2006 | new CPV-2a |
| FJ432717.1/08-5-WH | Hubei/ Central China | 2008 | new CPV-2a |
| EU441280.1/PV/PL/HeN02/08 | Henan/ Central China | 2008 | CPV-2a |
| KC881278.1/CPV-2b-wuhan2 | Hubei/ Central China | 2010 | new CPV-2b |
| JX120178.1/CPV-GZ | Guangdong/ South China | 2010 | new CPV-2a |
| KJ438799.1/Henan12 | Henan/ Central China | 2011 | new CPV-2a |
| KJ438804.1/Henan38 | Henan/ Central China | 2012 | new CPV-2a |
| KM924289.1/Henan32 | Henan/ Central China | 2013 | ew CPV-2a |
| KJ438805.1/Henan42 | Henan/ Central China | 2013 | new CPV-2a |
| MF467242.1/CPV-GX1581 | Guangxi/ South China | 2015 | CPV-2c |
| MF467241.1/CPV-HN1503 | Hainan/ South China | 2015 | new CPV-2a |
| MF467239.1/CPV-HN1519 | Hainan/ South China | 2015 | new CPV-2b |
| MF467229.1/CPV-HN1617 | Hainan/ South China | 2016 | CPV-2c |
| KF482478.1/1130 | South china | 2009 | new CPV-2b |
| KF482477.1/S10 | South china | 2009 | new CPV-2a |
| KF482476.1/S9 | South china | 2009 | new CPV-2a |
| KF482475.1/S8 | South china | 2009 | new CPV-2a |
| KF482474.1/S6 | South china | 2009 | new CPV-2a |
| KF482473.1/S4 | South china | 2009 | new CPV-2a |
| KF482472.1/S3 | South china | 2009 | new CPV-2a |
| KF482471.1/G15 | South china | 2009 | new CPV-2b |
| KF482470.1/G13 | South china | 2009 | new CPV-2a |
| KF482469.1/G5 | South china | 2009 | new CPV-2a |
| KF482468.1/G1 | South china | 2009 | new CPV-2b |
| JF767494.1/S7 | South china | 2009 | new CPV-2a |
| JF767493.1/S5 | South china | 2009 | new CPV-2a |
| JF767492.1/S2 | South china | 2009 | new CPV-2a |
| GU569937.1/GZ0202 | Guizhou/ Southwest China | 2002 | new CPV-2b |
| JQ996152.1/YAZA4 | Sichuan/ Southwest China | 2010 | new CPV-2a |
| JQ996151.1/YAZA2 | Sichuan/ Southwest China | 2010 | new CPV-2a |
| HQ651237.1/YAZA1 | Sichuan/ Southwest China | 2010 | new CPV-2a |
| GU569944.1/GZ0201 | Guizhou/ Southwest China | 2002 | new CPV-2b |
| GU569940.1/YN0203 | Yunnan/ Southwest China | 2002 | new CPV-2b |
| GU569939.1/YN0202 | Yunnan/ Southwest China | 2002 | new CPV-2a |
| GU569938.1/YN0201 | Yunnan/ Southwest China | 2002 | new CPV-2b |
| FJ435345.1/CPV-04/08/CN-4 | Sichuan/ Southwest China | 2008 | new CPV-2a |
| JQ996155.1/LSZA1 | Sichuan/ Southwest China | 2010 | new CPV-2a |
| JQ996154.1/CDZA1 | Sichuan/ Southwest China | 2010 | new CPV-2a |
| JQ996153.1/YAZA5 | Sichuan/ Southwest China | 2011 | new CPV-2a |
| KT156825/ MDJ-1 | Mudanjiang /Northeast China | 2014 | new CPV-2a |
| KT156826/ MDJ-7 | Mudanjiang /Northeast China | 2014 | new CPV-2a |
| KT156827/ MDJ-9 | Mudanjiang /Northeast China | 2014 | new CPV-2a |
| KT156828/ MDJ-15 | Mudanjiang /Northeast China | 2014 | new CPV-2a |
| KT156829/MDJ-20 | Mudanjiang /Northeast China | 2015 | new CPV-2a |
| KT156830/ MDJ-22 | Mudanjiang /Northeast China | 2015 | new CPV-2a |
| KT156831/ HRB-A4 | Harbin/Northeast China | 2014 | new CPV-2b |
| KT156832/ HRB-A6 | Harbin/Northeast China | 2014 | CPV-2c |
| KT156833/ HRB-b2 | Harbin/Northeast China | 2014 | new CPV-2b |
| KT156833/ HRB-b2 | Harbin/Northeast China | 2014 | new CPV-2b |
| KT156834/ HRB-e2 | Harbin/Northeast China | 2015 | new CPV-2a |
| KT156835/ HRB-ee7 | Harbin/Northeast China | 2014 | new CPV-2a |
| KT156836/ HRB-F8 | Harbin/Northeast China | 2014 | new CPV-2a |
| KT156837/ HRB-J8 | Harbin/Northeast China | 2014 | new CPV-2b |
| GQ169549/ bj-5 | Beijing/North China | 2010 | new CPV-2a |
| KJ674820/ Wu | Wuhan/Central China | 2013 | new CPV-2a |
| GQ169539/ Wh-3 | Wuhan/Central China | 2009 | new CPV-2a |
| GQ169550/ 1-nj | Nanjing/East China | 2009 | new CPV-2a |
| JX121627/ CPVSH-3/2011 | Shanghai/ East China | 2011 | new CPV-2a |
| KR611470/ CPV HLJ-14-1 | Heilongjiang/Northeast China | 2014 | new CPV-2a |
| KR611471/ CPV HLJ-14-2 | Heilongjiang/Northeast China | 2014 | new CPV-2b |
| KR611472/ CPV HLJ-14-3 | Heilongjiang/Northeast China | 2014 | new CPV-2a |
| KR611473/ CPV HLJ-14-4 | Heilongjiang/Northeast China | 2014 | new CPV-2a |
| KR611474/ CPV HLJ-14-5 | Heilongjiang/Northeast China | 2014 | new CPV-2b |
| KR611475/ CPV HLJ-14-6 | Heilongjiang/Northeast China | 2014 | new CPV-2a |
| KR611476/ CPV HLJ-14-7 | Heilongjiang/Northeast China | 2014 | new CPV-2b |
| KR611477/ CPV HLJ-14-8 | Heilongjiang/Northeast China | 2014 | new CPV-2b |
| KR611478/ CPV HLJ-14-9 | Heilongjiang/Northeast China | 2014 | new CPV-2a |
| KR611479/ CPV HLJ-14-10 | Heilongjiang/Northeast China | 2014 | new CPV-2a |
| KR611479/ CPV HLJ-14-10 | Heilongjiang/Northeast China | 2014 | new CPV-2a |
| KR611480/ CPV HLJ-14-11 | Heilongjiang/Northeast China | 2014 | new CPV-2a |
| KR611481/ CPV HLJ-14-12 | Heilongjiang/Northeast China | 2014 | new CPV-2a |
| KR611482/ CPV HLJ-14-13 | Heilongjiang/Northeast China | 2014 | new CPV-2b |
| KR611483/ CPV HLJ-14-14 | Heilongjiang/Northeast China | 2014 | new CPV-2a |
| KR611484/ CPV HLJ-14-15 | Heilongjiang/Northeast China | 2014 | new CPV-2a |
| KR611485/ CPV HLJ-14-16 | Heilongjiang/Northeast China | 2014 | new CPV-2a |
| KR611486/ CPV HLJ-14-17 | Heilongjiang/Northeast China | 2014 | new CPV-2a |
| KR611487/ CPV HLJ-14-18 | Heilongjiang/Northeast China | 2014 | new CPV-2a |
| KR611488/CPV JL-14-1 | Jilin/Northeast China | 2014 | new CPV-2a |
| KR611489/CPV JL-14-2 | Jilin/Northeast China | 2014 | new CPV-2a |
| KR611490/CPV JL-14-3 | Jilin/Northeast China | 2014 | new CPV-2b |
| KR611491/CPV JL-14-4 | Jilin/Northeast China | 2014 | new CPV-2a |
| KR611492/CPV JL-14-5 | Jilin/Northeast China | 2014 | new CPV-2b |
| KR611493/CPV JL-14-6 | Jilin/Northeast China | 2014 | new CPV-2b |
| KR611494/CPV JL-14-7 | Jilin/Northeast China | 2014 | new CPV-2a |
| KR611495/CPV JL-14-8 | Jilin/Northeast China | 2014 | new CPV-2a |
| KR611496/CPV JL-14-9 | Jilin/Northeast China | 2014 | new CPV-2a |
| KR611497/CPV JL-14-10 | Jilin/Northeast China | 2014 | new CPV-2a |
| KR611498/CPV LN-14-1 | Liaoning/Northeast China | 2014 | new CPV-2a |
| KR611499/CPV LN-14-2 | Liaoning/Northeast China | 2014 | new CPV-2a |
| KR611500/CPV LN-14-3 | Liaoning/Northeast China | 2014 | new CPV-2b |
| KR611501/CPV LN-14-4 | Liaoning/Northeast China | 2014 | new CPV-2a |
| KR611502/CPV LN-14-5 | Liaoning/Northeast China | 2014 | new CPV-2b |
| KR611503/CPV LN-14-6 | Liaoning/Northeast China | 2014 | new CPV-2a |
| KR611504/CPV LN-14-7 | Liaoning/Northeast China | 2014 | new CPV-2b |
| KR611505/CPV LN-14-8 | Liaoning/Northeast China | 2014 | new CPV-2a |
| KR611506/CPV LN-14-9 | Liaoning/Northeast China | 2014 | new CPV-2a |
| KR611507/CPV LN-14-10 | Liaoning/Northeast China | 2014 | new CPV-2a |
| KR611508/CPV LN-14-11 | Liaoning/Northeast China | 2014 | new CPV-2a |
| KR611509/CPV LN-14-12 | Liaoning/Northeast China | 2014 | new CPV-2a |
| KR611510/CPV LN-14-13 | Liaoning/Northeast China | 2014 | new CPV-2b |
| KR611459/CPV HB-14-1 | Hebei/North China | 2014 | new CPV-2b |
| KR611460/CPV HB-14-2 | Hebei/North China | 2014 | new CPV-2b |
| KR611461/CPV HB-14-3 | Hebei/North China | 2014 | new CPV-2b |
| KR611462/CPV HB-14-4 | Hebei/North China | 2014 | new CPV-2b |
| KR611463/CPV HB-14-5 | Hebei/North China | 2014 | new CPV-2b |
| KR611464/CPV HB-14-6 | Hebei/North China | 2014 | new CPV-2b |
| KR611465/CPV HB-14-7 | Hebei/North China | 2014 | new CPV-2a |
| KR611466/CPV HB-14-8 | Hebei/North China | 2014 | new CPV-2b |
| KR611467/CPV HB-14-9 | Hebei/North China | 2014 | new CPV-2b |
| KR611468/CPV HB-14-10 | Hebei/North China | 2014 | new CPV-2a |
| KR611469/CPV HB-14-11 | Hebei/North China | 2014 | new CPV-2b |
| KR611511/CPV SD-14-1 | Shandong/East China | 2014 | new CPV-2a |
| KR611512/CPV SD-14-2 | Shandong/East China | 2014 | new CPV-2a |
| KR611513/CPV SD-14-3 | Shandong/East China | 2014 | new CPV-2a |
| KR611514/CPV SD-14-4 | Shandong/East China | 2014 | new CPV-2a |
| KR611515/CPV SD-14-5 | Shandong/East China | 2014 | new CPV-2a |
| KR611516/CPV SD-14-6 | Shandong/East China | 2014 | new CPV-2a |
| KR611517/CPV SD-14-7 | Shandong/East China | 2014 | new CPV-2a |
| KR611518/CPV SD-14-8 | Shandong/East China | 2014 | new CPV-2a |
| KR611519/CPV SD-14-9 | Shandong/East China | 2014 | new CPV-2a |
| KR6115110/CPV SD-14-10 | Shandong/East China | 2014 | new CPV-2b |
| KR6115111/CPV SD-14-11 | Shandong/East China | 2014 | new CPV-2a |
| KR6115112/CPV SD-14-12 | Shandong/East China | 2014 | CPV-2c |
| KF676668/ CPV JS2 | Nanjing/East China | 2009 | new CPV-2a |
| EF666063/ CPV BJ015/07 | Beijing/North China | 2007 | new CPV-2a |
| EF666066/ CPV BJ018/07 | Beijing/North China | 2007 | new CPV-2a |
| EF011664/CPV-B-2004 | Beijing/North China | 2004 | new CPV-2a |
| DQ354068/ RPPV | Yunnan/Soutewest China | 2004 | new CPV-2a |
| EU095252/ CPV-NJ-04 | Nanjing/East China | 2004 | new CPV-2a |
| EU310373/ CPV/nj01/06 | Nanjing/East China | 2006 | new CPV-2a |
| EF666059/ CPV/BJ004/07 | Beijing/North China | 2007 | new CPV-2a |
| EF666062/ CPV/BJ010/07 | Beijing/North China | 2007 | new CPV-2a |
| EU145959/ CPV/BJ077/07 | Beijing/North China | 2007 | new CPV-2a |
| EU145958/ CPV/BJ069/07 | Beijing/North China | 2007 | new CPV-2a |
| EU145960/CPV/BJ082/07 | Beijing/North China | 2007 | new CPV-2a |
| EU170352/ CPV-SHZ | Shihezi/Northwest China | 2007 | new CPV-2a |
| EU697385/ CPV-HLJ-JQ | Heilongjiang/Northeast China | 2007 | new CPV-2a |
| EU666060/ CPV/BJ005/07 | Beijing/North China | 2007 | new CPV-2a |
| FJ432717/CPV-08-5-WH | Wuhan/Central China | 2008 | new CPV-2a |
| DQ903936/ SCDN | Sichuan/Southwest China | 2005 | new CPV-2a |
| EF028071/ SCJM | Sichuan/Southwest China | 2005 | new CPV-2b |
| FJ435343/CN080402 | Sichuan/Southwest China | 2005 | new CPV-2a |
| FJ435346/ CN080405 | Sichuan/Southwest China | 2005 | new CPV-2a |
| KC556927/CPV-JS1 | Nanjing/East China | 2009 | new CPV-2a |
| KT074278/ HRB-A5 | Harbin/Northeast China | 2014 | new CPV-2a |
| KT074280/ HRB-A8 | Harbin/Northeast China | 2014 | new CPV-2a |
| KT074279/HRB-B0 | Harbin/Northeast China | 2014 | new CPV-2a |
| KT074283/HRB-B7 | Harbin/Northeast China | 2014 | new CPV-2a |
| KT074318/HRB-b9 | Harbin/Northeast China | 2014 | new CPV-2a |
| KT074285/HRB-C2 | Harbin/Northeast China | 2014 | new CPV-2a |
| KT074286/HRB-C5 | Harbin/Northeast China | 2014 | new CPV-2a |
| KT074287/HRB-C6 | Harbin/Northeast China | 2014 | new CPV-2a |
| KT074289/HRB-C9 | Harbin/Northeast China | 2014 | new CPV-2a |
| KT074290/HRB-b5 | Harbin/Northeast China | 2014 | new CPV-2a |
| KT074281/HRB-bb7 | Harbin/Northeast China | 2014 | new CPV-2a |
| KT074284/HRB-bb8 | Harbin/Northeast China | 2014 | new CPV-2a |
| KT074291/HRB-dd2 | Harbin/Northeast China | 2014 | new CPV-2a |
| KT074294/HRB-D4 | Harbin/Northeast China | 2014 | new CPV-2a |
| KT074295/HRB-D8 | Harbin/Northeast China | 2014 | new CPV-2a |
| KT074296/HRB-D9 | Harbin/Northeast China | 2014 | new CPV-2a |
| KT074297/HRB-E0 | Harbin/Northeast China | 2014 | new CPV-2a |
| KT074298/HRB-E1 | Harbin/Northeast China | 2014 | new CPV-2a |
| KT074299/HRB-e2 | Harbin/Northeast China | 2015 | new CPV-2a |
| KT074300/HRB-E8 | Harbin/Northeast China | 2014 | new CPV-2a |
| KT074301/HRB-F0 | Harbin/Northeast China | 2015 | new CPV-2a |
| KT074302/HRB-F4 | Harbin/Northeast China | 2014 | new CPV-2a |
| KT074305/HRB-G4 | Harbin/Northeast China | 2015 | new CPV-2a |
| KT074307/HRB-H2 | Harbin/Northeast China | 2014 | new CPV-2a |
| KT074308/HRB-I1 | Harbin/Northeast China | 2014 | new CPV-2a |
| KT074309/HRB-I3 | Harbin/Northeast China | 2014 | new CPV-2a |
| KT074310/HRB-I4 | Harbin/Northeast China | 2014 | new CPV-2a |
| KT074312/HRB-I8 | Harbin/Northeast China | 2015 | new CPV-2a |
| KT074313/HRB-J1 | Harbin/Northeast China | 2014 | new CPV-2a |
| KT074316/HRB-K1 | Harbin/Northeast China | 2015 | new CPV-2a |
| KT074317/HRB-K3 | Harbin/Northeast China | 2015 | new CPV-2a |
| KT074304/HRB-G0 | Harbin/Northeast China | 2014 | new CPV-2a |
| KT074303/HRB-F8 | Harbin/Northeast China | 2014 | new CPV-2a |
| KT074259/MDJ-7 | Mudanjiang/Northeast China | 2014 | new CPV-2a |
| KT074260/MDJ-9 | Mudanjiang/Northeast China | 2014 | new CPV-2a |
| KT074261/MDJ-15 | Mudanjiang/Northeast China | 2014 | new CPV-2a |
| KT074262/MDJ-18 | Mudanjiang/Northeast China | 2015 | new CPV-2a |
| KT074263/MDJ-20 | Mudanjiang/Northeast China | 2015 | new CPV-2a |
| KT074265/MDJ-22 | Mudanjiang/Northeast China | 2014 | new CPV-2a |
| KT074267/MDJ-28 | Mudanjiang/Northeast China | 2014 | new CPV-2a |
| KT074268/MDJ-29 | Mudanjiang/Northeast China | 2015 | new CPV-2a |
| KT074269/MDJ-32 | Mudanjiang/Northeast China | 2015 | new CPV-2a |
| KT074272/MDJ-40 | Mudanjiang/Northeast China | 2015 | new CPV-2a |
| KT074273/DQ-alpha1 | Daqing/Northeast China | 2014 | new CPV-2a |
| KT074274/DQ-beta1 | Daqing/Northeast China | 2015 | new CPV-2a |
| KT074264/MDJ-21 | Mudanjiang/Northeast China | 2014 | new CPV-2a |
| KT074266/MDJ-27 | Mudanjiang/Northeast China | 2015 | new CPV-2a |
| KT074270/MDJ-33 | Mudanjiang/Northeast China | 2015 | new CPV-2a |
| KT074271/MDJ-37 | Mudanjiang/Northeast China | 2015 | new CPV-2a |
| KT074275/DQ-beta7 | Daqing/Northeast China | 2015 | new CPV-2a |
| KT074277/HRB-A2 | Harbin/Northeast China | 2014 | new CPV-2a |
| KT074282/HRB-B8 | Harbin/Northeast China | 2014 | new CPV-2a |
| KT074288/HRB-C7 | Harbin/Northeast China | 2014 | new CPV-2a |
| KT074276/HRB-a1 | Harbin/Northeast China | 2014 | new CPV-2a |
| KT074292/HRB-ee7 | Harbin/Northeast China | 2014 | new CPV-2a |
| KT074293/HRB-D2 | Harbin/Northeast China | 2014 | new CPV-2a |
| KT074306/HRB-G6 | Harbin/Northeast China | 2014 | new CPV-2a |
| KT074311/HRB-I7 | Harbin/Northeast China | 2014 | new CPV-2a |
| KT074314/HRB-J4 | Harbin/Northeast China | 2014 | new CPV-2a |
| KT074315/HRB-J7 | Harbin/Northeast China | 2014 | new CPV-2a |
| KT074336/DQ-beta0 | Daqing/Northeast China | 2014 | new CPV-2b |
| KT074337/ DQ-beta4 | Daqing/Northeast China | 2014 | new CPV-2b |
| KT074338/DQ-beta9 | Daqing/Northeast China | 2015 | new CPV-2b |
| KT074319/HRB-A4 | Harbin/Northeast China | 2014 | new CPV-2b |
| KT074320/HRB-A9 | Harbin/Northeast China | 2014 | new CPV-2b |
| KT074321/HRB-B6 | Harbin/Northeast China | 2014 | new CPV-2b |
| KT074322/HRB-aa8 | Harbin/Northeast China | 2014 | new CPV-2b |
| KT074323/HRB-b2 | Harbin/Northeast China | 2014 | new CPV-2b |
| KT074324/ HRB-d1 | Harbin/Northeast China | 2014 | new CPV-2b |
| KT074325/HRB-d3 | Harbin/Northeast China | 2014 | new CPV-2b |
| KT074326/HRB-d5 | Harbin/Northeast China | 2014 | new CPV-2b |
| KT074327/HRB-E7 | Harbin/Northeast China | 2014 | new CPV-2b |
| KT074328/HRB-F5 | Harbin/Northeast China | 2014 | new CPV-2b |
| KT074329/HRB-F6 | Harbin/Northeast China | 2014 | new CPV-2b |
| KT074330/HRB-F7 | Harbin/Northeast China | 2014 | new CPV-2b |
| KT074331/HRB-H3 | Harbin/Northeast China | 2015 | new CPV-2b |
| KT074332/HRB-H4 | Harbin/Northeast China | 2015 | new CPV-2b |
| KT074333/HRB-H8 | Harbin/Northeast China | 2015 | new CPV-2b |
| KT074334/HRB-I5 | Harbin/Northeast China | 2015 | new CPV-2b |
| KT074335/HRB-J8 | Harbin/Northeast China | 2015 | new CPV-2b |
| KT074348/MDJ-4 | Mudanjiang/Northeast China | 2015 | CPV-2c |
| KT074349/MDJ-6 | Mudanjiang/Northeast China | 2014 | CPV-2c |
| KT074350/MDJ-11 | Mudanjiang/Northeast China | 2014 | CPV-2c |
| KT074351/MDJ-24 | Mudanjiang/Northeast China | 2014 | CPV-2c |
| KT074352/MDJ-39 | Mudanjiang/Northeast China | 2015 | CPV-2c |
| KT074339/HRB-A6 | Harbin/Northeast China | 2014 | CPV-2c |
| KT074340/ HRB-aa9 | Harbin/Northeast China | 2014 | CPV-2c |
| KT074341/HRB-b3 | Harbin/Northeast China | 2014 | CPV-2c |
| KT074342/HRB-E5 | Harbin/Northeast China | 2014 | CPV-2c |
| KT074343/HRB-e6 | Harbin/Northeast China | 2015 | CPV-2c |
| KT074344/HRB-E9 | Harbin/Northeast China | 2014 | CPV-2c |
| KT074345/HRB-F9 | Harbin/Northeast China | 2015 | CPV-2c |
| KT074346/HRB-G8 | Harbin/Northeast China | 2014 | CPV-2c |
| KT074347/HRB-J6 | Harbin/Northeast China | 2014 | CPV-2c |
| KF638400/CPV-s5 | South China | 2011 | new CPV-2a |
| JQ268284/CPV-LZ2 | Northwest China | 2011 | new CPV-2b |
| JQ268283/CPV-LZ1 | Northwest China | 2011 | new CPV-2a |
| KF803589/ BJ-A1 | Beijing/North China | 2010 | new CPV-2a |
| KF803590/ BJ-A2 | Beijing/North China | 2010 | new CPV-2a |
| KF803591/ BJ-A45 | Beijing/North China | 2010 | new CPV-2a |
| KF803592/ BJ-A48 | Beijing/North China | 2010 | new CPV-2a |
| KF803593/ BJ-A49 | Beijing/North China | 2010 | new CPV-2a |
| KF803594/ BJ-A50 | Beijing/North China | 2010 | new CPV-2a |
| KF803595/ BJ-A53 | Beijing/North China | 2010 | new CPV-2a |
| KF803596/ BJ-A57 | Beijing/North China | 2010 | new CPV-2a |
| KF803597/ BJ-A61 | Beijing/North China | 2010 | new CPV-2a |
| KF803598/ BJ-A63 | Beijing/North China | 2010 | new CPV-2b |
| KF803599/ BJ-A64 | Beijing/North China | 2010 | new CPV-2a |
| KF803600/ BJ-A68 | Beijing/North China | 2010 | new CPV-2a |
| KF803601/ BJ-A69 | Beijing/North China | 2010 | new CPV-2a |
| KF803602/ BJ-A72 | Beijing/North China | 2010 | new CPV-2a |
| KF803603/ BJ-A108 | Beijing/North China | 2010 | new CPV-2b |
| KF803604/ BJ-B4 | Beijing/North China | 2011 | new CPV-2a |
| KF803605/ BJ-B5 | Beijing/North China | 2011 | new CPV-2a |
| KF803606/ BJ-B6 | Beijing/North China | 2011 | new CPV-2b |
| KF803607/ BJ-B8 | Beijing/North China | 2011 | new CPV-2a |
| KF803608/ BJ-B10 | Beijing/North China | 2011 | new CPV-2a |
| KF803609/ BJ-B11 | Beijing/North China | 2011 | new CPV-2a |
| KF803610/ BJ-B13 | Beijing/North China | 2011 | new CPV-2a |
| KF803611/ BJ-B16 | Beijing/North China | 2011 | new CPV-2b |
| KF803612/ BJ-B19 | Beijing/North China | 2011 | new CPV-2a |
| KF803613/ BJ-B21 | Beijing/North China | 2011 | new CPV-2a |
| KF803614/ BJ-B22 | Beijing/North China | 2011 | new CPV-2a |
| KF803615/ BJ-B25 | Beijing/North China | 2011 | new CPV-2a |
| KF803616/ BJ- B26 | Beijing/North China | 2011 | new CPV-2a |
| KF803617/ BJ-B28 | Beijing/North China | 2011 | new CPV-2a |
| KF803618/ BJ-B31 | Beijing/North China | 2011 | new CPV-2a |
| KF803619/ BJ-B32 | Beijing/North China | 2011 | new CPV-2a |
| KF803620/ BJ-B33 | Beijing/North China | 2011 | new CPV-2a |
| KF803621/ BJ-B34 | Beijing/North China | 2011 | new CPV-2a |
| KF803622/ BJ-B38 | Beijing/North China | 2011 | new CPV-2a |
| KF803623/ BJ-B39 | Beijing/North China | 2011 | new CPV-2a |
| KF803624/ BJ-B40 | Beijing/North China | 2011 | new CPV-2a |
| KF803625/ BJ-B41 | Beijing/North China | 2011 | new CPV-2a |
| KF803626/ BJ-B42 | Beijing/North China | 2011 | new CPV-2a |
| KF803627/ BJ-B43 | Beijing/North China | 2011 | new CPV-2b |
| KF803628/ BJ-B44 | Beijing/North China | 2011 | new CPV-2a |
| KF803629/ BJ-D4 | Beijing/North China | 2012 | new CPV-2a |
| KF803630/ BJ-D6 | Beijing/North China | 2012 | new CPV-2a |
| KF803631/ BJ-D14 | Beijing/North China | 2012 | new CPV-2a |
| KF803632/ BJ-D15 | Beijing/North China | 2012 | new CPV-2a |
| KF803633/ BJ-E4 | Beijing/North China | 2012 | new CPV-2a |
| KF803634/ BJ-E13 | Beijing/North China | 2012 | new CPV-2a |
| KF803635/ BJ-E14 | Beijing/North China | 2012 | new CPV-2a |
| KF803636/ BJ-E34 | Beijing/North China | 2012 | new CPV-2a |
| KF803637/ BJ-E53 | Beijing/North China | 2012 | new CPV-2a |
| KF803638/ BJ-E64 | Beijing/North China | 2012 | new CPV-2a |
| KF803639/ BJ-E81 | Beijing/North China | 2012 | new CPV-2a |
| KF803640/ BJ-P21 | Beijing/North China | 2013 | new CPV-2a |
| KF803641/ BJ-P27 | Beijing/North China | 2013 | new CPV-2a |
| KF803642/ BJ-P33 | Beijing/North China | 2013 | new CPV-2a |
| KF803643/ BJ-P34 | Beijing/North China | 2013 | new CPV-2a |
| KT162006/CPV BJ14-11 | Beijing/North China | 2014 | CPV-2c |
| KT162007/CPV BJ14-12 | Beijing/North China | 2014 | CPV-2c |
| KT162008/CPV BJ14-13 | Beijing/North China | 2014 | CPV-2c |
| KT162009/CPV BJ14-17 | Beijing/North China | 2014 | CPV-2c |
| KT162010/CPV BJ14-18 | Beijing/North China | 2014 | CPV-2c |
| KT162011/CPV BJ14-20 | Beijing/North China | 2014 | CPV-2c |
| KT162012/CPV BJ14-21 | Beijing/North China | 2014 | CPV-2c |
| KT162013/CPV BJ14-22 | Beijing/North China | 2014 | CPV-2c |
| KT162014/CPV BJ14-32 | Beijing/North China | 2014 | CPV-2c |
| KT162015/CPV BJ14-33 | Beijing/North China | 2014 | CPV-2c |
| KT162016/CPV BJ14-34 | Beijing/North China | 2014 | CPV-2c |
| KT162017/CPV BJ14-35 | Beijing/North China | 2014 | CPV-2c |
| KT162018/CPV BJ14-36 | Beijing/North China | 2014 | CPV-2c |
| KT162019/CPV BJ14-37 | Beijing/North China | 2014 | CPV-2c |
| KT162020/CPV BJ14-38 | Beijing/North China | 2014 | CPV-2c |
| KT162021/CPV BJ14-39 | Beijing/North China | 2014 | CPV-2c |
| KT162022/CPV BJ14-1 | Beijing/North China | 2014 | New CPV-2b |
| KT162023/ CPV BJ14-2 | Beijing/North China | 2014 | New CPV-2b |
| KT162024/CPV BJ15-2 | Beijing/North China | 2014 | New CPV-2b |
| KT162025/CPV BJ15-7 | Beijing/North China | 2014 | New CPV-2b |
| KT162026/CPV BJ15-11 | Beijing/North China | 2014 | New CPV-2b |
| KT162027/CPV BJ15-12 | Beijing/North China | 2014 | New CPV-2b |
| KT162028/CPV BJ15-13 | Beijing/North China | 2014 | New CPV-2b |
| KT162029/CPV BJ14-5 | Beijing/North China | 2014 | New CPV-2a |
| KT162030/CPV BJ14-6 | Beijing/North China | 2014 | New CPV-2a |
| KT162031/CPV BJ14-7 | Beijing/North China | 2014 | New CPV-2a |
| KT162032/CPV BJ14-14 | Beijing/North China | 2014 | New CPV-2a |
| KT162033/CPV BJ14-16 | Beijing/North China | 2014 | New CPV-2a |
| KT162034/CPV BJ14-23 | Beijing/North China | 2014 | New CPV-2a |
| KT162035/CPV BJ14-24 | Beijing/North China | 2014 | New CPV-2a |
| KT162036/CPV BJ14-26 | Beijing/North China | 2014 | New CPV-2a |
| KT162037/CPV BJ14-27 | Beijing/North China | 2014 | New CPV-2a |
| KT162038/CPV BJ14-28 | Beijing/North China | 2014 | New CPV-2a |
| KT162039/CPV BJ15-4 | Beijing/North China | 2014 | New CPV-2a |
| KT162040/CPV BJ15-3 | Beijing/North China | 2014 | New CPV-2a |
| KT162041/CPV BJ15-1 | Beijing/North China | 2014 | New CPV-2a |
| KT162042/CPV BJ15-5 | Beijing/North China | 2014 | New CPV-2a |
| KT162043/CPV BJ15-6 | Beijing/North China | 2014 | New CPV-2a |
| KT162044/CPV BJ15-9 | Beijing/North China | 2014 | New CPV-2a |
| KT162045/CPV BJ15-14 | Beijing/North China | 2014 | New CPV-2a |
| EU145954/CPV/BJ044/O7 | Beijing/North China | 2007 | new CPV-2b |
| FJ435342/CPV-04/08/CN-1 | Sichuan/Southwest China | 2008 | CPV-2 |
| FJ435348/CPV-04/08/CN-7 | Sichuan/Southwest China | 2008 | CPV-2 |
| DQ177497/HN-3 | South (Guangdong) | 2010 | new CPV-2b |
| KF803615/2011-BJ-B25/ | Beijing/North China | 2011 | CPV-2a |
| KR002793/CPV/CN/HB1 | Hebei/North China | 2013 | new CPV-2b |
| KR002794/CPV/CN/HB3 | Hebei/North China | 2013 | new CPV-2a |
| KR002795/CPV/CN/JL1 | Jilin/Northeast China | 2013 | new CPV-2a |
| KR002796/CPV/CN/JL3 | Jilin/Northeast China | 2013 | new CPV-2a |
| KR002797/CPV/CN/JL4 | Jilin/Northeast China | 2013 | new CPV-2a |
| KR002798/CPV/CN/JL5 | Jilin/Northeast China | 2013 | new CPV-2a |
| KR002799/CPV/CN/JL6 | Jilin/Northeast China | 2013 | new CPV-2b |
| KR002792/CPV/CN/SH1 | Shanghai/East China | 2013 | new CPV-2a |
| KR002800/CPV/CN/LN1 | Liaoning/Northeast China | 2014 | new CPV-2a |
| KR002801/CPV/CN/SD6 | Shandong/East China | 2014 | new CPV-2a |
| KR002802/CPV/CN/SD9 | Shandong/East China | 2014 | new CPV-2a |
| KR002803/CPV/CN/SD10 | Shandong/East China | 2014 | new CPV-2a |
| KR002804/CPV/CN/SD18 | Shandong/East China | 2014 | new CPV-2a |
| KR002805/CPV/CN/SD19 | Shandong/East China | 2014 | new CPV-2a |
| FJ435344/CPV-04/08 /CN-3 | Sichuan/Southwest China | 2008 | CPV-2 |
| KT156832/HRB-A6 | Harbin/Northeast China | 2014 | CPV-2c |
| KT162005/BJ14-8 | Beijing/North China | 2014 | CPV-2c |

**TABLE S4.** CPV-2 strains used in temporal distribution analysis.

| GenBank accession no./Name | Isolated time | Genotype |
| --- | --- | --- |
| GU392244.1/HB7 | 2009 | CPV-2 |
| GU392243.1/HB9 | 2009 | CPV-2 |
| GU392242.1/HB10 | 2009 | CPV-2 |
| GU392241.1/HB1 | 2009 | CPV-2 |
| GU392240.1/HB3 | 2009 | CPV-2 |
| GU392239.1/HB6 | 2009 | CPV-2 |
| MH177317.1/GX307 | 2017 | CVP-2c |
| MH177316.1/GX308 | 2017 | CVP-2c |
| MH177315.1/GX310 | 2017 | CVP-2c |
| MH177314.1/GX302 | 2017 | CVP-2c |
| MH177313.1/GX303 | 2017 | CVP-2c |
| MH177312.1/GX305 | 2017 | CVP-2c |
| MH177310.1/GX324 | 2017 | CVP-2c |
| MH177309.1/GX349 | 2017 | CVP-2c |
| MH177308.1/GX356 | 2017 | CVP-2c |
| MH177307.1/GX357 | 2017 | CVP-2c |
| MH177306.1/GX372 | 2017 | CVP-2c |
| MH177305.1/GX373 | 2017 | CVP-2c |
| MH177304.1/GX337 | 2017 | CVP-2c |
| MH177303.1/GX345 | 2017 | CVP-2c |
| MH177302.1/GX326 | 2017 | new CPV-2a |
| MH177301.1/GX304 | 2017 | new CPV-2a |
| MF996333.1/RDPV-DP2 | 2016 | new CPV-2a |
| MF996332.1/RDPV-DP1 | 2016 | new CPV-2a |
| GU380301.1/04/09 | 2009 | new CPV-2a |
| GU380299.1/02/09 | 2009 | new CPV-2b |
| GU380298.1/01/09 | 2009 | new CPV-2a |
| KF482478.1/1130 | 2009 | new CPV-2b |
| KF482477.1/S10 | 2009 | new CPV-2a |
| KF482476.1/S9 | 2009 | new CPV-2a |
| KF482475.1/S8 | 2009 | new CPV-2a |
| KF482474.1/S6 | 2009 | new CPV-2a |
| KF482473.1/S4 | 2009 | new CPV-2a |
| KF482472.1/S3 | 2009 | new CPV-2a |
| KF482471.1/G15 | 2009 | new CPV-2b |
| KF482470.1/G13 | 2009 | new CPV-2a |
| KF482469.1/G5 | 2009 | new CPV-2a |
| KF482468.1/G1 | 2009 | new CPV-2b |
| GU569945.1/QB0101 | 2001 | new CPV-2a |
| GU569941.1/SD0201 | 2002 | new CPV-2a |
| GU569937.1/GZ0202 | 2002 | new CPV-2b |
| FJ432716.1/06-11-NJ | 2006 | new CPV-2a |
| KP260509.1/BJ14-9 | 2014 | CPV-2c |
| KR869678.1/CPV/BJ524 | 2014 | new CPV-2b |
| KR869677.1/CPV/BJ480 | 2014 | new CPV-2b |
| KR869676.1/CPV/BJ361 | 2014 | new CPV-2b |
| KR869675.1/CPV/BJ355 | 2014 | new CPV-2b |
| KR869674.1/CPV/BJ339 | 2014 | new CPV-2b |
| KR869673.1/CPV/BJ279 | 2014 | new CPV-2b |
| KR869672.1/CPV/BJ219 | 2014 | new CPV-2b |
| KR869671.1/CPV/BJ137 | 2014 | new CPV-2b |
| KR869670.1/CPV/BJ131 | 2014 | new CPV-2b |
| KR869669.1/CPV/BJ104 | 2014 | new CPV-2b |
| KR869668.1/CPV/BJ450 | 2014 | new CPV-2a |
| KR869667.1/CPV/BJ439 | 2014 | new CPV-2a |
| KR869666.1/CPV/BJ326 | 2014 | new CPV-2a |
| KR869665.1/CPV/BJ301 | 2014 | new CPV-2a |
| KR869664.1/CPV/BJ135 | 2014 | new CPV-2a |
| KR869663.1/CPV/BJ67 | 2014 | new CPV-2a |
| KR869662.1/CPV/BJ59 | 2014 | new CPV-2a |
| KR869661.1/CPV/BJ57 | 2014 | new CPV-2a |
| KR869660.1/CPV/BJ49 | 2014 | new CPV-2a |
| KR869659.1/CPV/BJ30 | 2014 | new CPV-2a |
| KR869658.1/CPV/BJ25 | 2014 | new CPV-2a |
| KR869657.1/CPV/BJ24 | 2014 | new CPV-2a |
| KR869656.1/CPV/BJ23 | 2014 | new CPV-2a |
| KR869655.1/CPV/BJ14 | 2014 | new CPV-2a |
| KR869654.1/CPV/BJ13 | 2014 | new CPV-2a |
| KR869653.1/CPV/BJ09 | 2014 | new CPV-2a |
| KR869652.1/CPV/BJ01 | 2014 | new CPV-2a |
| JQ743906.1/CPV-2(10) | 2010 | new CPV-2a |
| JQ743905.1/CPV-5(10) | 2010 | new CPV-2a |
| JQ743904.1/CPV-6(10) | 2010 | new CPV-2a |
| JQ743903.1/CPV-7(10) | 2010 | new CPV-2a |
| JQ743902.1/CPV-13(10) | 2010 | new CPV-2a |
| JQ743901.1/CPV-HT(11) | 2011 | new CPV-2a |
| JQ743900.1/CPV-LBLD2(11) | 2011 | new CPV-2a |
| JQ743899.1/CPV-SS2(11) | 2011 | new CPV-2a |
| JQ743898.1/CPV-SS3(11) | 2011 | new CPV-2a |
| JQ743896.1/CPV-JQ4(11) | 2011 | new CPV-2a |
| JQ743894.1/CPV-BM(11) | 2011 | new CPV-2b |
| JQ743893.1/CPV-HS(11) | 2011 | new CPV-2b |
| JQ743892.1/CPV-BG(11) | 2011 | new CPV-2b |
| JQ743891.1/CPV-10(10) | 2010 | new CPV-2b |
| JQ743890.1/CPV-4(10) | 2010 | new CPV-2b |
| JN403045.1/Shanaxi | 2011 | new CPV-2a |
| JF767494.1/S7 | 2009 | new CPV-2a |
| JF767493.1/S5 | 2009 | new CPV-2a |
| JF767492.1/S2 | 2009 | new CPV-2a |
| GU452715.1 /11/09 | 2009 | new CPV-2a |
| GU452714.1/10/09 | 2009 | new CPV-2a |
| GU452713.1/09/09 | 2009 | new CPV-2a |
| EU441279.1/PV/PL/HeN03/08 | 2008 | new CPV-2a |
| MF001439.1/CPV/CN/YZ5 | 2016 | new CPV-2a |
| MF001438.1/CPV/CN/YZ4 | 2016 | new CPV-2a |
| MF001437.1/CPV/CN/YZ8 | 2016 | CPV-2c |
| MF001436.1/CPV/CN/YZ3 | 2016 | CPV-2c |
| MF001435.1/CPV/CN/YZ1 | 2016 | CPV-2c |
| JQ996152.1/YAZA4/2010 | 2010 | new CPV-2a |
| JQ996151.1/YAZA2 | 2010 | new CPV-2a |
| HQ651237.1/YAZA1 | 2010 | new CPV-2a |
| EU377537.1/CPV/WH02/06 | 2006 | new CPV-2a |
| JQ743895.1/CPV-JM3(11) | 2011 | new CPV-2a |
| GU569943.1/YB8301 | 1983 | CPV-2 |
| GU569948.1/CC8601 | 1986 | CPV-2a |
| GU569947.1/BJ9901 | 1999 | new CPV-2a |
| GU569946.1/JL0201 | 2001 | new CPV-2a |
| GU569944.1/GZ0201 | 2002 | new CPV-2b |
| GU569942.1/JL0202 | 2002 | new CPV-2a |
| GU569940.1/YN0203 | 2002 | new CPV-2b |
| GU569939.1/YN0202 | 2002 | new CPV-2a |
| GU569938.1/YN0201 | 2002 | new CPV-2b |
| GQ857599.1/CPV05-04 | 2005 | new CPV-2b |
| GQ857602.1/CPV06-03 | 2005 | new CPV-2b |
| GQ857601.1/CPV06-02 | 2006 | new CPV-2b |
| GQ857608.1/CPV07-06 | 2007 | new CPV-2b |
| GQ857607.1/CPV07-05 | 2007 | new CPV-2a |
| EU145954/CPV/BJ044/O7 | 2007 | new CPV-2b |
| EU483510/CPV-JB3 | 2007 | new CPV-2b |
| EU483517/CPV-ZD3 | 2007 | new CPV-2b |
| GQ857613.1/CPV08-05 | 2008 | new CPV-2a |
| GQ857610.1/CPV08-02 | 2008 | new CPV-2b |
| FJ432717.1/08-5-WH | 2008 | new CPV-2a |
| GU569936.1/CNJL0804 | 2008 | new CPV-2a |
| EU441280.1/PV/PL/HeN02/08 | 2008 | CPV-2a |
| KJ186145.1/NJ07-2 | 2008 | new CPV-2a |
| KJ186143.1/JLHD08-1 | 2008 | new CPV-2a |
| KJ186142.1/JLDAAN08-2 | 2008 | new CPV-2a |
| FJ432718.1/CPV-Cv | 2008 | CPV-2 |
| FJ435345.1/CPV-04/08/CN-4 | 2008 | new CPV-2a |
| FJ435342/CPV-04/08/CN-1 | 2008 | CPV-2 |
| FJ435348/CPV-04/08/CN-7 | 2008 | CPV-2 |
| FJ265781/CPV307/TW05 | 2008 | CPV-2b |
| GU380304.1/07/09 | 2009 | new CPV-2a |
| GU380303.1/06/09 | 2009 | CPV-2c |
| GU380302.1/05/09 | 2009 | new CPV-2a |
| GU380300.1/03/09 | 2009 | new CPV-2b |
| GU380305.1/08/09 | 2009 | CPV-2c |
| KC881278.1/CPV-2b-wuhan2 | 2010 | new CPV-2b |
| KM083041.1/RDPV-HeB(10)9 | 2010 | CPV-2 |
| JX120178.1/CPV-GZ | 2010 | new CPV-2a |
| JF789638.1/BD-1010 | 2010 | new CPV-2a |
| JQ996155.1/LSZA1 | 2010 | new CPV-2a |
| JQ996154.1/CDZA1 | 2010 | new CPV-2a |
| HQ883273.1/BJ/2010 | 2010 | new CPV-2a |
| DQ177497/HN-3 | 2010 | new CPV-2b |
| JQ996153.1/YAZA5 | 2011 | new CPV-2a |
| JX121624.1/BJ-2 | 2011 | new CPV-2a |
| KJ438799.1/Henan12/2011 | 2011 | new CPV-2a |
| KF803615/2011-BJ-B25/ | 2011 | CPV-2a |
| KC262178.1/MPCPV-SX | 2012 | CPV-2a |
| KJ438804.1/Henan38 | 2012 | new CPV-2a |
| KM924289.1/Henan32 | 2013 | ew CPV-2a |
| KJ438805.1/Henan42 | 2013 | new CPV-2a |
| KR002793/CPV/CN/HB1 | 2013 | new CPV-2b |
| KR002794/CPV/CN/HB3 | 2013 | new CPV-2a |
| KR002795/CPV/CN/JL1 | 2013 | new CPV-2a |
| KR002796/CPV/CN/JL3 | 2013 | new CPV-2a |
| KR002797/CPV/CN/JL4 | 2013 | new CPV-2a |
| KR002798/CPV/CN/JL5 | 2013 | new CPV-2a |
| KR002799/CPV/CN/JL6 | 2013 | new CPV-2b |
| KR002792/CPV/CN/SH1 | 2013 | new CPV-2a |
| KR002800/CPV/CN/LN1 | 2014 | new CPV-2a |
| KR002801/CPV/CN/SD6 | 2014 | new CPV-2a |
| KR002802/CPV/CN/SD9 | 2014 | new CPV-2a |
| KR002803/CPV/CN/SD10 | 2014 | new CPV-2a |
| KR002804/CPV/CN/SD18 | 2014 | new CPV-2a |
| KR002805/CPV/CN/SD19 | 2014 | new CPV-2a |
| FJ435344/CPV-04/08 /CN-3 | 2008 | CPV-2 |
| KT156832/HRB-A6 | 2014 | CPV-2c |
| KT162005/BJ14-8 | 2014 | CPV-2c |
| KR611522.1/CPV-SD-14-12 | 2014 | CPV-2c |
| KR611521.1/CPV-SD-14-11 | 2014 | new CPV-2a |
| KR611520.1/CPV-SD-14-10 | 2014 | new CPV-2b |
| KR611510.1/CPV-LN-14-13 | 2014 | new CPV-2b |
| KR611509.1/CPV-LN-14-12 | 2014 | new CPV-2a |
| KR611490.1/CPV-JL-14-3 | 2014 | new CPV-2b |
| KR611489.1/CPV-JL-14-2 | 2014 | new CPV-2a |
| KR611487.1/CPV-HLJ-14-18 | 2014 | new CPV-2a |
| KR611482.1/CPV-HLJ-14-13 | 2014 | new CPV-2b |
| KR611468.1/CPV-HB-14-10 | 2014 | new CPV-2a |
| KR611467.1/CPV-HB-14-9 | 2014 | new CPV-2b |
| KT162037.1/BJ14-27 | 2014 | new CPV-2a |
| KT162021.1/BJ14-39 | 2014 | CPV-2c |
| KP749855.1/YANJI-2 | 2014 | new CPV-2b |
| KP749854.1/YANJI-1 | 2014 | CPV-2c |
| KP749853.1/WANGQING-3 | 2014 | new CPV-2a |
| KP749852.1/WANGQING-2 | 2014 | new CPV-2b |
| KP749851.1/WANGQING-1 | 2014 | CPV-2c |
| KP749850.1/TUMEN-2 | 2014 | new CPV-2a |
| KP749848.1/LONGJING-2 | 2014 | new CPV-2a |
| KP749847.1/LONGJING-1 | 2014 | new CPV-2b |
| KP749846.1/HUNCHUN-3 | 2014 | new CPV-2a |
| KP749843.1/HELONG-2 | 2014 | new CPV-2a |
| KP749841.1/DUNHUA-3 | 2014 | new CPV-2a |
| KP749840.1/DUNHUA-2 | 2014 | new CPV-2b |
| KP749838.1/ANTU-2 | 2014 | new CPV-2a |
| MF467242.1/CPV-GX1581 | 2015 | CPV-2c |
| MF467241.1/CPV-HN1503 | 2015 | new CPV-2a |
| MF467239.1/CPV-HN1519 | 2015 | new CPV-2b |
| KU244254/NPUST014/2015 | 2015 | CPV-2c |
| MF467226.1//CPV-JS1591 | 2015 | new CPV-2a |
| MF467225.1/CPV-JS1592 | 2015 | CPV-2c |
| MF467224.1/CPV-ZJ1579 | 2015 | new CPV-2a |
| KT162046.1/BJ15-15 | 2015 | new CPV-2a |
| KT162028.1/BJ15-13 | 2015 | new CPV-2b |
| MF996334.1/RDPV-DP3 VP2 | 2016 | CPV-2 |
| MF467229.1/CPV-HN1617 | 2016 | CPV-2c |
| KT156825/ MDJ-1 | 2014 | new CPV-2a |
| KT156826/ MDJ-7 | 2014 | new CPV-2a |
| KT156827/ MDJ-9 | 2014 | new CPV-2a |
| KT156828/ MDJ-15 | 2014 | new CPV-2a |
| KT156829/MDJ-20 | 2015 | new CPV-2a |
| KT156830/ MDJ-22 | 2015 | new CPV-2a |
| KT156831/ HRB-A4 | 2014 | new CPV-2b |
| KT156832/ HRB-A6 | 2014 | CPV-2c |
| KT156833/ HRB-b2 | 2014 | new CPV-2b |
| KT156833/ HRB-b2 | 2014 | new CPV-2b |
| KT156834/ HRB-e2 | 2015 | new CPV-2a |
| KT156835/ HRB-ee7 | 2014 | new CPV-2a |
| KT156836/ HRB-F8 | 2014 | new CPV-2a |
| KT156837/ HRB-J8 | 2014 | new CPV-2b |
| GQ169549/ bj-5 | 2010 | new CPV-2a |
| KJ674820/ Wu | 2013 | new CPV-2a |
| GQ169539/ Wh-3 | 2009 | new CPV-2a |
| GQ169550/ 1-nj | 2009 | new CPV-2a |
| JX121627/ CPVSH-3/2011 | 2011 | new CPV-2a |
| KR611470/ CPV HLJ-14-1 | 2014 | new CPV-2a |
| KR611471/ CPV HLJ-14-2 | 2014 | new CPV-2b |
| KR611472/ CPV HLJ-14-3 | 2014 | new CPV-2a |
| KR611473/ CPV HLJ-14-4 | 2014 | new CPV-2a |
| KR611474/ CPV HLJ-14-5 | 2014 | new CPV-2b |
| KR611475/ CPV HLJ-14-6 | 2014 | new CPV-2a |
| KR611476/ CPV HLJ-14-7 | 2014 | new CPV-2b |
| KR611477/ CPV HLJ-14-8 | 2014 | new CPV-2b |
| KR611478/ CPV HLJ-14-9 | 2014 | new CPV-2a |
| KR611479/ CPV HLJ-14-10 | 2014 | new CPV-2a |
| KR611479/ CPV HLJ-14-10 | 2014 | new CPV-2a |
| KR611480/ CPV HLJ-14-11 | 2014 | new CPV-2a |
| KR611481/ CPV HLJ-14-12 | 2014 | new CPV-2a |
| KR611482/ CPV HLJ-14-13 | 2014 | new CPV-2b |
| KR611483/ CPV HLJ-14-14 | 2014 | new CPV-2a |
| KR611484/ CPV HLJ-14-15 | 2014 | new CPV-2a |
| KR611485/ CPV HLJ-14-16 | 2014 | new CPV-2a |
| KR611486/ CPV HLJ-14-17 | 2014 | new CPV-2a |
| KR611487/ CPV HLJ-14-18 | 2014 | new CPV-2a |
| KR611488/CPV JL-14-1 | 2014 | new CPV-2a |
| KR611489/CPV JL-14-2 | 2014 | new CPV-2a |
| KR611490/CPV JL-14-3 | 2014 | new CPV-2b |
| KR611491/CPV JL-14-4 | 2014 | new CPV-2a |
| KR611492/CPV JL-14-5 | 2014 | new CPV-2b |
| KR611493/CPV JL-14-6 | 2014 | new CPV-2b |
| KR611494/CPV JL-14-7 | 2014 | new CPV-2a |
| KR611495/CPV JL-14-8 | 2014 | new CPV-2a |
| KR611496/CPV JL-14-9 | 2014 | new CPV-2a |
| KR611497/CPV JL-14-10 | 2014 | new CPV-2a |
| KR611498/CPV LN-14-1 | 2014 | new CPV-2a |
| KR611499/CPV LN-14-2 | 2014 | new CPV-2a |
| KR611500/CPV LN-14-3 | 2014 | new CPV-2b |
| KR611501/CPV LN-14-4 | 2014 | new CPV-2a |
| KR611502/CPV LN-14-5 | 2014 | new CPV-2b |
| KR611503/CPV LN-14-6 | 2014 | new CPV-2a |
| KR611504/CPV LN-14-7 | 2014 | new CPV-2b |
| KR611505/CPV LN-14-8 | 2014 | new CPV-2a |
| KR611506/CPV LN-14-9 | 2014 | new CPV-2a |
| KR611507/CPV LN-14-10 | 2014 | new CPV-2a |
| KR611508/CPV LN-14-11 | 2014 | new CPV-2a |
| KR611509/CPV LN-14-12 | 2014 | new CPV-2a |
| KR611510/CPV LN-14-13 | 2014 | new CPV-2b |
| KR611459/CPV HB-14-1 | 2014 | new CPV-2b |
| KR611460/CPV HB-14-2 | 2014 | new CPV-2b |
| KR611461/CPV HB-14-3 | 2014 | new CPV-2b |
| KR611462/CPV HB-14-4 | 2014 | new CPV-2b |
| KR611463/CPV HB-14-5 | 2014 | new CPV-2b |
| KR611464/CPV HB-14-6 | 2014 | new CPV-2b |
| KR611465/CPV HB-14-7 | 2014 | new CPV-2a |
| KR611466/CPV HB-14-8 | 2014 | new CPV-2b |
| KR611467/CPV HB-14-9 | 2014 | new CPV-2b |
| KR611468/CPV HB-14-10 | 2014 | new CPV-2a |
| KR611469/CPV HB-14-11 | 2014 | new CPV-2b |
| KR611511/CPV SD-14-1 | 2014 | new CPV-2a |
| KR611512/CPV SD-14-2 | 2014 | new CPV-2a |
| KR611513/CPV SD-14-3 | 2014 | new CPV-2a |
| KR611514/CPV SD-14-4 | 2014 | new CPV-2a |
| KR611515/CPV SD-14-5 | 2014 | new CPV-2a |
| KR611516/CPV SD-14-6 | 2014 | new CPV-2a |
| KR611517/CPV SD-14-7 | 2014 | new CPV-2a |
| KR611518/CPV SD-14-8 | 2014 | new CPV-2a |
| KR611519/CPV SD-14-9 | 2014 | new CPV-2a |
| KR6115110/CPV SD-14-10 | 2014 | new CPV-2b |
| KR6115111/CPV SD-14-11 | 2014 | new CPV-2a |
| KR6115112/CPV SD-14-12 | 2014 | CPV-2c |
| KF676668/ CPV JS2 | 2009 | new CPV-2a |
| EF666063/ CPV BJ015/07 | 2007 | new CPV-2a |
| EF666066/ CPV BJ018/07 | 2007 | new CPV-2a |
| EF011664/CPV-B-2004 | 2004 | new CPV-2a |
| DQ354068/ RPPV | 2004 | new CPV-2a |
| EU095252/ CPV-NJ-04 | 2004 | new CPV-2a |
| EU310373/ CPV/nj01/06 | 2006 | new CPV-2a |
| EF666059/ CPV/BJ004/07 | 2007 | new CPV-2a |
| EF666062/ CPV/BJ010/07 | 2007 | new CPV-2a |
| EU145959/ CPV/BJ077/07 | 2007 | new CPV-2a |
| EU145958/ CPV/BJ069/07 | 2007 | new CPV-2a |
| EU145960/CPV/BJ082/07 | 2007 | new CPV-2a |
| EU170352/ CPV-SHZ | 2007 | new CPV-2a |
| EU697385/ CPV-HLJ-JQ | 2007 | new CPV-2a |
| EU666060/ CPV/BJ005/07 | 2007 | new CPV-2a |
| FJ432717/CPV-08-5-WH | 2008 | new CPV-2a |
| DQ903936/ SCDN | 2005 | new CPV-2a |
| EF028071/ SCJM | 2005 | new CPV-2b |
| FJ435343/CN080402 | 2005 | new CPV-2a |
| FJ435346/ CN080405 | 2005 | new CPV-2a |
| KC556927/CPV-JS1 | 2009 | new CPV-2a |
| KT074278/ HRB-A5 | 2014 | new CPV-2a |
| KT074280/ HRB-A8 | 2014 | new CPV-2a |
| KT074279/HRB-B0 | 2014 | new CPV-2a |
| KT074283/HRB-B7 | 2014 | new CPV-2a |
| KT074318/HRB-b9 | 2014 | new CPV-2a |
| KT074285/HRB-C2 | 2014 | new CPV-2a |
| KT074286/HRB-C5 | 2014 | new CPV-2a |
| KT074287/HRB-C6 | 2014 | new CPV-2a |
| KT074289/HRB-C9 | 2014 | new CPV-2a |
| KT074290/HRB-b5 | 2014 | new CPV-2a |
| KT074281/HRB-bb7 | 2014 | new CPV-2a |
| KT074284/HRB-bb8 | 2014 | new CPV-2a |
| KT074291/HRB-dd2 | 2014 | new CPV-2a |
| KT074294/HRB-D4 | 2014 | new CPV-2a |
| KT074295/HRB-D8 | 2014 | new CPV-2a |
| KT074296/HRB-D9 | 2014 | new CPV-2a |
| KT074297/HRB-E0 | 2014 | new CPV-2a |
| KT074298/HRB-E1 | 2014 | new CPV-2a |
| KT074299/HRB-e2 | 2015 | new CPV-2a |
| KT074300/HRB-E8 | 2014 | new CPV-2a |
| KT074301/HRB-F0 | 2015 | new CPV-2a |
| KT074302/HRB-F4 | 2014 | new CPV-2a |
| KT074305/HRB-G4 | 2015 | new CPV-2a |
| KT074307/HRB-H2 | 2014 | new CPV-2a |
| KT074308/HRB-I1 | 2014 | new CPV-2a |
| KT074309/HRB-I3 | 2014 | new CPV-2a |
| KT074310/HRB-I4 | 2014 | new CPV-2a |
| KT074312/HRB-I8 | 2015 | new CPV-2a |
| KT074313/HRB-J1 | 2014 | new CPV-2a |
| KT074316/HRB-K1 | 2015 | new CPV-2a |
| KT074317/HRB-K3 | 2015 | new CPV-2a |
| KT074304/HRB-G0 | 2014 | new CPV-2a |
| KT074303/HRB-F8 | 2014 | new CPV-2a |
| KT074259/MDJ-7 | 2014 | new CPV-2a |
| KT074260/MDJ-9 | 2014 | new CPV-2a |
| KT074261/MDJ-15 | 2014 | new CPV-2a |
| KT074262/MDJ-18 | 2015 | new CPV-2a |
| KT074263/MDJ-20 | 2015 | new CPV-2a |
| KT074265/MDJ-22 | 2014 | new CPV-2a |
| KT074267/MDJ-28 | 2014 | new CPV-2a |
| KT074268/MDJ-29 | 2015 | new CPV-2a |
| KT074269/MDJ-32 | 2015 | new CPV-2a |
| KT074272/MDJ-40 | 2015 | new CPV-2a |
| KT074273/DQ-alpha1 | 2014 | new CPV-2a |
| KT074274/DQ-beta1 | 2015 | new CPV-2a |
| KT074264/MDJ-21 | 2014 | new CPV-2a |
| KT074266/MDJ-27 | 2015 | new CPV-2a |
| KT074270/MDJ-33 | 2015 | new CPV-2a |
| KT074271/MDJ-37 | 2015 | new CPV-2a |
| KT074275/DQ-beta7 | 2015 | new CPV-2a |
| KT074277/HRB-A2 | 2014 | new CPV-2a |
| KT074282/HRB-B8 | 2014 | new CPV-2a |
| KT074288/HRB-C7 | 2014 | new CPV-2a |
| KT074276/HRB-a1 | 2014 | new CPV-2a |
| KT074292/HRB-ee7 | 2014 | new CPV-2a |
| KT074293/HRB-D2 | 2014 | new CPV-2a |
| KT074306/HRB-G6 | 2014 | new CPV-2a |
| KT074311/HRB-I7 | 2014 | new CPV-2a |
| KT074314/HRB-J4 | 2014 | new CPV-2a |
| KT074315/HRB-J7 | 2014 | new CPV-2a |
| KT074336/DQ-beta0 | 2014 | new CPV-2b |
| KT074337/ DQ-beta4 | 2014 | new CPV-2b |
| KT074338/DQ-beta9 | 2015 | new CPV-2b |
| KT074319/HRB-A4 | 2014 | new CPV-2b |
| KT074320/HRB-A9 | 2014 | new CPV-2b |
| KT074321/HRB-B6 | 2014 | new CPV-2b |
| KT074322/HRB-aa8 | 2014 | new CPV-2b |
| KT074323/HRB-b2 | 2014 | new CPV-2b |
| KT074324/ HRB-d1 | 2014 | new CPV-2b |
| KT074325/HRB-d3 | 2014 | new CPV-2b |
| KT074326/HRB-d5 | 2014 | new CPV-2b |
| KT074327/HRB-E7 | 2014 | new CPV-2b |
| KT074328/HRB-F5 | 2014 | new CPV-2b |
| KT074329/HRB-F6 | 2014 | new CPV-2b |
| KT074330/HRB-F7 | 2014 | new CPV-2b |
| KT074331/HRB-H3 | 2015 | new CPV-2b |
| KT074332/HRB-H4 | 2015 | new CPV-2b |
| KT074333/HRB-H8 | 2015 | new CPV-2b |
| KT074334/HRB-I5 | 2015 | new CPV-2b |
| KT074335/HRB-J8 | 2015 | new CPV-2b |
| KT074348/MDJ-4 | 2015 | CPV-2c |
| KT074349/MDJ-6 | 2014 | CPV-2c |
| KT074350/MDJ-11 | 2014 | CPV-2c |
| KT074351/MDJ-24 | 2014 | CPV-2c |
| KT074352/MDJ-39 | 2015 | CPV-2c |
| KT074339/HRB-A6 | 2014 | CPV-2c |
| KT074340/ HRB-aa9 | 2014 | CPV-2c |
| KT074341/HRB-b3 | 2014 | CPV-2c |
| KT074342/HRB-E5 | 2014 | CPV-2c |
| KT074343/HRB-e6 | 2015 | CPV-2c |
| KT074344/HRB-E9 | 2014 | CPV-2c |
| KT074345/HRB-F9 | 2015 | CPV-2c |
| KT074346/HRB-G8 | 2014 | CPV-2c |
| KT074347/HRB-J6 | 2014 | CPV-2c |
| KF638400/CPV-s5 | 2011 | new CPV-2a |
| JQ268284/CPV-LZ2 | 2011 | new CPV-2b |
| JQ268283/CPV-LZ1 | 2011 | new CPV-2a |
| KF803589/ BJ-A1 | 2010 | new CPV-2a |
| KF803590/ BJ-A2 | 2010 | new CPV-2a |
| KF803591/ BJ-A45 | 2010 | new CPV-2a |
| KF803592/ BJ-A48 | 2010 | new CPV-2a |
| KF803593/ BJ-A49 | 2010 | new CPV-2a |
| KF803594/ BJ-A50 | 2010 | new CPV-2a |
| KF803595/ BJ-A53 | 2010 | new CPV-2a |
| KF803596/ BJ-A57 | 2010 | new CPV-2a |
| KF803597/ BJ-A61 | 2010 | new CPV-2a |
| KF803598/ BJ-A63 | 2010 | new CPV-2b |
| KF803599/ BJ-A64 | 2010 | new CPV-2a |
| KF803600/ BJ-A68 | 2010 | new CPV-2a |
| KF803601/ BJ-A69 | 2010 | new CPV-2a |
| KF803602/ BJ-A72 | 2010 | new CPV-2a |
| KF803603/ BJ-A108 | 2010 | new CPV-2b |
| KF803604/ BJ-B4 | 2011 | new CPV-2a |
| KF803605/ BJ-B5 | 2011 | new CPV-2a |
| KF803606/ BJ-B6 | 2011 | new CPV-2b |
| KF803607/ BJ-B8 | 2011 | new CPV-2a |
| KF803608/ BJ-B10 | 2011 | new CPV-2a |
| KF803609/ BJ-B11 | 2011 | new CPV-2a |
| KF803610/ BJ-B13 | 2011 | new CPV-2a |
| KF803611/ BJ-B16 | 2011 | new CPV-2b |
| KF803612/ BJ-B19 | 2011 | new CPV-2a |
| KF803613/ BJ-B21 | 2011 | new CPV-2a |
| KF803614/ BJ-B22 | 2011 | new CPV-2a |
| KF803615/ BJ-B25 | 2011 | new CPV-2a |
| KF803616/ BJ- B26 | 2011 | new CPV-2a |
| KF803617/ BJ-B28 | 2011 | new CPV-2a |
| KF803618/ BJ-B31 | 2011 | new CPV-2a |
| KF803619/ BJ-B32 | 2011 | new CPV-2a |
| KF803620/ BJ-B33 | 2011 | new CPV-2a |
| KF803621/ BJ-B34 | 2011 | new CPV-2a |
| KF803622/ BJ-B38 | 2011 | new CPV-2a |
| KF803623/ BJ-B39 | 2011 | new CPV-2a |
| KF803624/ BJ-B40 | 2011 | new CPV-2a |
| KF803625/ BJ-B41 | 2011 | new CPV-2a |
| KF803626/ BJ-B42 | 2011 | new CPV-2a |
| KF803627/ BJ-B43 | 2011 | new CPV-2b |
| KF803628/ BJ-B44 | 2011 | new CPV-2a |
| KF803629/ BJ-D4 | 2012 | new CPV-2a |
| KF803630/ BJ-D6 | 2012 | new CPV-2a |
| KF803631/ BJ-D14 | 2012 | new CPV-2a |
| KF803632/ BJ-D15 | 2012 | new CPV-2a |
| KF803633/ BJ-E4 | 2012 | new CPV-2a |
| KF803634/ BJ-E13 | 2012 | new CPV-2a |
| KF803635/ BJ-E14 | 2012 | new CPV-2a |
| KF803636/ BJ-E34 | 2012 | new CPV-2a |
| KF803637/ BJ-E53 | 2012 | new CPV-2a |
| KF803638/ BJ-E64 | 2012 | new CPV-2a |
| KF803639/ BJ-E81 | 2012 | new CPV-2a |
| KF803640/ BJ-P21 | 2013 | new CPV-2a |
| KF803641/ BJ-P27 | 2013 | new CPV-2a |
| KF803642/ BJ-P33 | 2013 | new CPV-2a |
| KF803643/ BJ-P34 | 2013 | new CPV-2a |
| KT162006/CPV BJ14-11 | 2014 | CPV-2c |
| KT162007/CPV BJ14-12 | 2014 | CPV-2c |
| KT162008/CPV BJ14-13 | 2014 | CPV-2c |
| KT162009/CPV BJ14-17 | 2014 | CPV-2c |
| KT162010/CPV BJ14-18 | 2014 | CPV-2c |
| KT162011/CPV BJ14-20 | 2014 | CPV-2c |
| KT162012/CPV BJ14-21 | 2014 | CPV-2c |
| KT162013/CPV BJ14-22 | 2014 | CPV-2c |
| KT162014/CPV BJ14-32 | 2014 | CPV-2c |
| KT162015/CPV BJ14-33 | 2014 | CPV-2c |
| KT162016/CPV BJ14-34 | 2014 | CPV-2c |
| KT162017/CPV BJ14-35 | 2014 | CPV-2c |
| KT162018/CPV BJ14-36 | 2014 | CPV-2c |
| KT162019/CPV BJ14-37 | 2014 | CPV-2c |
| KT162020/CPV BJ14-38 | 2014 | CPV-2c |
| KT162021/CPV BJ14-39 | 2014 | CPV-2c |
| KT162022/CPV BJ14-1 | 2014 | New CPV-2b |
| KT162023/ CPV BJ14-2 | 2014 | New CPV-2b |
| KT162024/CPV BJ15-2 | 2014 | New CPV-2b |
| KT162025/CPV BJ15-7 | 2014 | New CPV-2b |
| KT162026/CPV BJ15-11 | 2014 | New CPV-2b |
| KT162027/CPV BJ15-12 | 2014 | New CPV-2b |
| KT162028/CPV BJ15-13 | 2014 | New CPV-2b |
| KT162029/CPV BJ14-5 | 2014 | New CPV-2a |
| KT162030/CPV BJ14-6 | 2014 | New CPV-2a |
| KT162031/CPV BJ14-7 | 2014 | New CPV-2a |
| KT162032/CPV BJ14-14 | 2014 | New CPV-2a |
| KT162033/CPV BJ14-16 | 2014 | New CPV-2a |
| KT162034/CPV BJ14-23 | 2014 | New CPV-2a |
| KT162035/CPV BJ14-24 | 2014 | New CPV-2a |
| KT162036/CPV BJ14-26 | 2014 | New CPV-2a |
| KT162037/CPV BJ14-27 | 2014 | New CPV-2a |
| KT162038/CPV BJ14-28 | 2014 | New CPV-2a |
| KT162039/CPV BJ15-4 | 2014 | New CPV-2a |
| KT162040/CPV BJ15-3 | 2014 | New CPV-2a |
| KT162041/CPV BJ15-1 | 2014 | New CPV-2a |
| KT162042/CPV BJ15-5 | 2014 | New CPV-2a |
| KT162043/CPV BJ15-6 | 2014 | New CPV-2a |
| KT162044/CPV BJ15-9 | 2014 | New CPV-2a |
| KT162045/CPV BJ15-14 | 2014 | New CPV-2a |
| FJ435342/CPV-04/08/CN-1 | 2008 | CPV-2 |
| FJ435348/CPV-04/08/CN-7 | 2008 | CPV-2 |
| FJ435344/CPV-04/08 /CN-3 | 2008 | CPV-2 |
